# Parts-Per-Million of Soluble Pd^0^ Catalyze the Semi-Hydrogenation Reaction of Alkynes to Alkenes

**DOI:** 10.1021/acs.joc.2c00616

**Published:** 2022-05-18

**Authors:** Jordi Ballesteros-Soberanas, Jose A. Carrasco, Antonio Leyva-Pérez

**Affiliations:** Instituto de Tecnología Química, Universitat Politècnica de València-Consejo Superior de Investigaciones Científicas, Avda. de los Naranjos s/n, 46022 Valencia, Spain

## Abstract

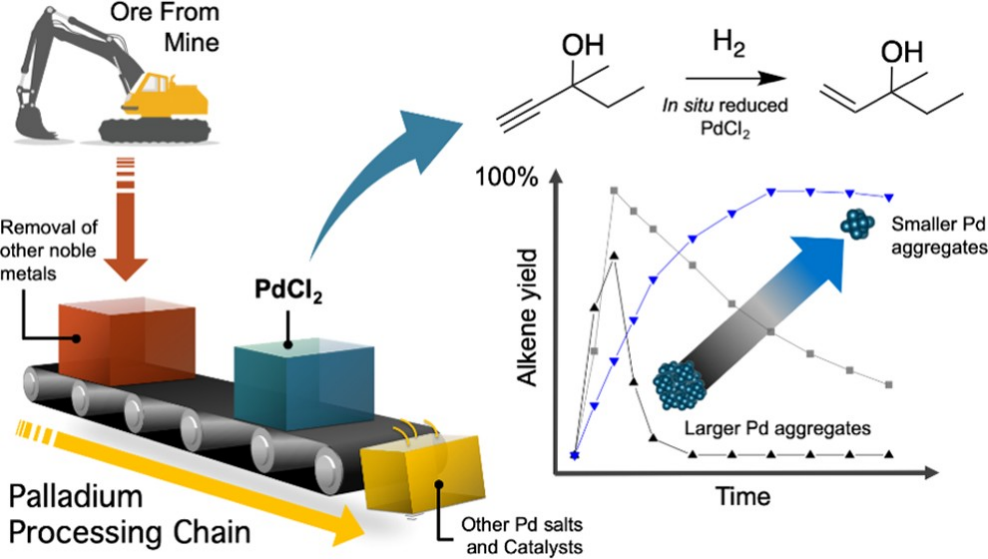

The synthesis of *cis*-alkenes is industrially carried out by selective semi-hydrogenation of alkynes with complex Pd catalysts, which include the Lindlar catalyst (PdPb on CaCO_3_) and c-Pd/TiS (colloidal ligand-protected Pd nanoparticles), among others. Here, we show that Pd^0^ atoms are generated from primary Pd salts (PdCl_2_, PdSO_4_, Pd(OH)_2_, PdO) with H_2_ in alcohol solutions, independently of the alkyne, to catalyze the semi-hydrogenation reaction with extraordinarily high efficiency (up to 735 s^–1^), yield (up to 99%), and selectivity (up to 99%). The easy-to-prepare Pd^0^ species hold other potential catalytic applications.

## Introduction

Pd catalysts are fundamental tools in modern industrial chemistry, with wide use in bulk to fine chemicals’ production, automotive emission control, and polymer chemistry, to name a few.^1−4^ However, the search for new catalysts is mainly focused on complex molecules and more sophisticated material engineering, of course scientifically rich and meritorious but, unfortunately, often difficult to prepare, expensive, and non applicable at the industrial scale.^5−15^ The opposite direction, i.e., the search of simplified Pd catalysts from primary Pd sources, is less explored.^16^ The appearance of single-atom catalysts (SACs) in the last years has come to somehow palliate this laborious catalyst design; however, the stabilization of Pd SACs generally requires a precise chemical environment, which translates into a complex synthesis of supporting solids.^17−20^ Thus, the use of cheaper and widely available Pd sources in catalysis remains a possibility worthy of study, also, considering the increasing price of this metal during the last few years, with no expectations to decrease.

The Pd-catalyzed selective semi-hydrogenation of alkynes to alkenes is an important reaction in industrial synthesis. On the one hand, this reaction is necessary for the purification of ethylene during polyethylene production, since the monomer stream contains variable amounts of acetylene, which must be hydrogenated to avoid the poisoning of the polymerization metal catalyst.^21,22^ The current alumina-supported Pd catalyst for acetylene semi-hydrogenation is a very complex system with more than five additives, despite chemo- or stereo-selectivity not being an issue here.^23^ On the other hand, the semi-hydrogenation reaction of the rest of the alkynes is considered the easiest way to prepare *cis*-alkenes, industrially utilized in the synthesis of nutraceuticals and vitamins, among other uses.^24^ The catalyst of choice is the classical Lindlar catalyst, composed of PdPb nanoparticles supported on CaCO_3_.^25^ A plethora of other Pd catalysts have been reported along the years, with the aim of increasing the catalytic efficiency of Pd and avoiding toxic Pb in their formulations, such as the commercially available colloidal Pd nanoparticles designed by BASF (c-Pd/TiS).^26^ However, these and the rest of the Pd catalysts reported in the open literature for the selective semi-hydrogenation of alkynes require an elaborated synthesis with increasing prices, where many of the starting Pd atoms are not ultimately productive.

Here, we show that Pd^0^ atoms (possibly coordinated with the solvent and/or reactants) are released in solution when simple Pd salts are treated with H_2_ in alcoholic solvents and that these Pd^0^ atoms catalyze the semi-hydrogenation reaction of alkynes to alkenes with a reaction rate far superior to any industrial catalysts, keeping the same level of selectivity to the *cis*-alkene. To our knowledge, these leached Pd^0^ atoms show some of the highest turnover numbers reported so far for the semi-hydrogenation reaction of alkynes, and the fact that they can be directly produced from primary Pd sources makes this catalytic system attractive.

## Results and Discussion

### Semi-Hydrogenation Kinetic Studies

**Figure 1:**
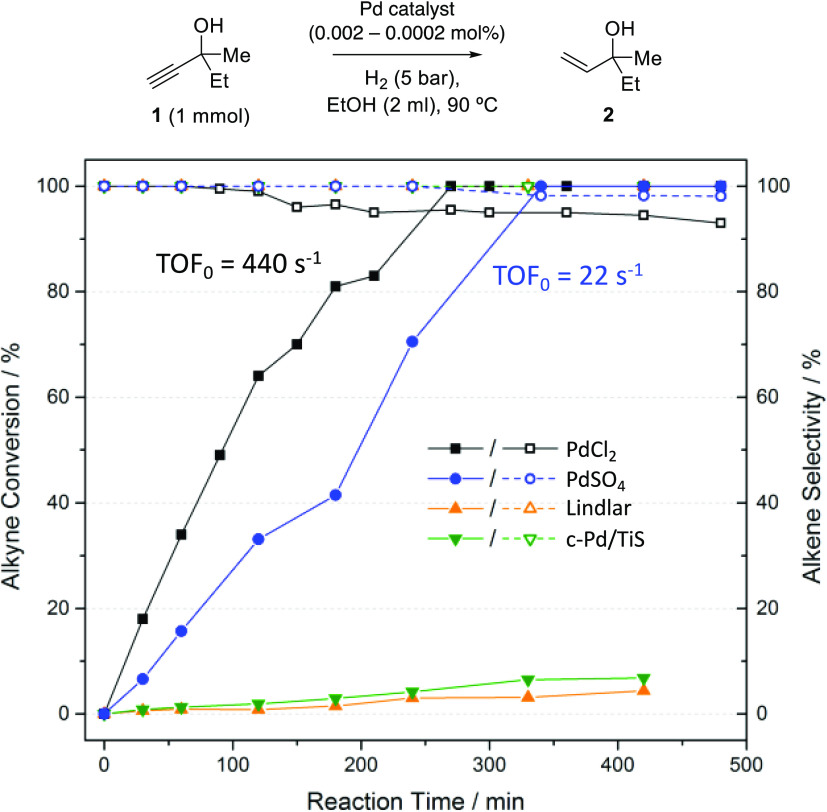
Kinetic plots for the hydrogenation of **1** to **2** with PdCl_2_, Lindlar′s and c-Pd/TiS catalysts (0.0002 mol %), and PdSO_4_ (0.002 mol %) in ethanol (0.5M) under 5 bar of H_2_ at 90 °C. The reactors were previously washed with *aqua regia*. Solid lines represent alkyne conversion, and dashed lines represent alkene selectivity; the remaining yields pertain to the corresponding alkane.

Our starting hypothesis is that simple Pd salts could evolve under alkyne hydrogenation reaction conditions to some kind of active Pd species.^19,27^Figure 1 shows the catalytic results for PdCl_2_, PdSO_4_, and, for the sake of comparison, also for the commercial Lindlar and c-Pd/TiS catalysts, using the hydrogenation of 3-methyl-1-pentyn-3-ol **1** as the model reaction. This reaction is of industrial interest as a synthetic step during the manufacturing of vitamins.^28,29^ PdCl_2_ catalyzes the synthesis of **2** with a yield and selectivity >95% and a remarkable initial turnover frequency (TOF_0_) = 440 s^–1^ at 90 °C reaction temperature, while the Lindlar and c-Pd/TiS catalysts show much lower yield and TOF_0_, even after prolonged reaction times. Being a reaction of industrial interest, a scaled-up reaction of 3 g of **1** has been performed at 90 °C, under 8 bar of H_2_. After 8 h, the reaction was stopped and full conversion was achieved, with a 97.5% yield of **2**. When the reaction was stopped at 18 h, only 20.1% of alkane was formed, the rest being product **2** (Figure S1). TOF_0_ was calculated by linear regression of the experimental points in the initial linear interval of the kinetic curve over the starting Pd amount (TOF_0_ = initial rate/total Pd amount). At lower temperatures (30 °C) and higher loadings, the catalytic performance of Pd salts is similar to that of the industrial catalysts (Figure S2).

The low loadings of PdCl_2_ require dilution in an inert solid (MgCO_3_) to weigh the needed amounts. Control experiments showed that MgCO_3_ does not have any influence on the catalytic action of PdCl_2_ (Figure S3) and, more importantly, that washings with *aqua regia* were required to completely remove Pd from batch to batch, since otherwise the tiny amounts of the insoluble Pd catalyst that remained in the reactor were active during the subsequent reactions and masked the catalytic activity of Pd for loadings lower than 0.04 mol % (Figures S4 and S5). Other Pd salts such as PdO, Pd(OH)_2_, and PdSO_4_ showed similar catalytic activity and selectivity to PdCl_2_ for the hydrogenation reaction of **1** (Figure S4). The general catalytic activity of simple Pd compounds at such low amounts must be taken in account by catalytic practitioners of this reaction, since Pd impurities^30^ can trigger the reaction and too much Pd amount decreases the overall efficiency.

**Figure 2:**
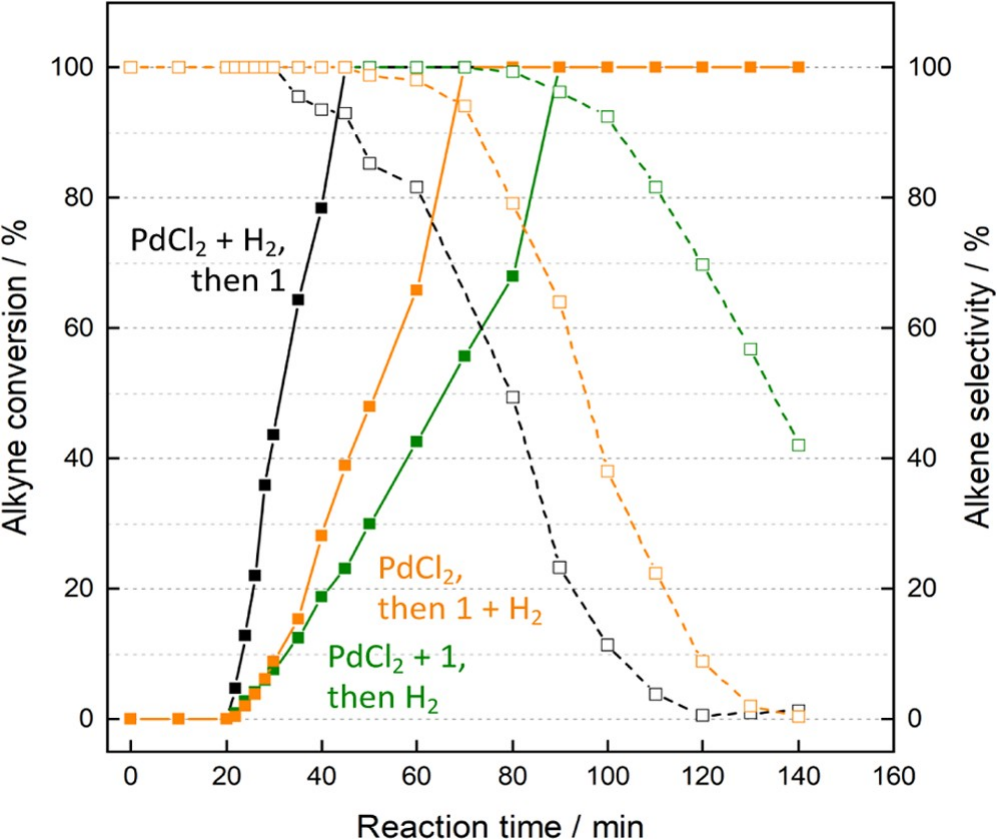
Kinetic plots for the hydrogenation of **1** to **2** with PdCl_2_ (0.04 mol %) in ethanol (0.5M), by adding **1** (black lines), H_2_ (green lines), or **1** + H_2_ (orange lines) after 10 min at 30 °C. The reactors were previously washed with *aqua regia*. Solid lines represent alkyne conversion, and dashed lines represent alkene selectivity; the remaining yields pertain to the corresponding alkane.

These results could point to a common catalytic Pd species generated *in situ* during the reaction. To shed light on this, three new experiments were designed, in which **1**, H_2_, or both **1** and H_2_ were not present at the beginning of the reaction but instead were added after 20 min. Figure 2 shows that the reaction starts immediately, without any induction time, when H_2_ is present from the beginning but not when PdCl_2_ and H_2_ have not been put together before, since a 10 min induction time is still observed. In other words, H_2_ seems to transform PdCl_2_ into a catalytically active Pd species in the ethanol solution without the action of alkyne **1**.

Different alcohols and water are suitable solvents for the reaction (Figure S8). The fact that tertiary alcohols are suitable for the reaction and that Fourier transform infrared spectroscopy (FTIR) experiments did not show any sign of dehydrogenation to aldehydes or ketones nor any related coupled product (Figure S9) discard the reducing action of boiling alcohols during the process.^31^ Furthermore, the higher boiling point solvent 2-methyl-2-butanol allows heating the hydrogenation reaction to 120 °C and achieving a TOF_0_ = 735 s^–1^ (2,600,000 h^–1^) with 0.0002 mol % PdCl_2_ loadings (Figure S10). It is difficult to find catalytic efficiencies of this order of magnitude in the open literature, even if we consider the leached Pd for calculations (∼0.00014 mol %, ∼4 million h^–1^, Table S1).

The hydrogenation of **1** catalyzed by PdCl_2_ (0.04 mol %) was repeated in the presence of 4 equivalents of PPh_3_ (0.16 mol %) at 30 °C, and a significant decrease of the catalytic activity was observed (Figure S11). In fact, we observed an inverse trend between the hydrogenation rates and the PPh_3_ equivalents with respect to the Pd in solution. Interestingly, the reaction rates decreased proportionally when the PPh_3_/Pd equivalents increased from 2:1 to 4:1, but remained invariant at higher or lower stoichiometries (Figure S12). *In situ* ultraviolet–visible absorption spectrophotometry (UV–vis) measurements of this reaction at room temperature showed the decrease of the PPh_3_ absorption band at 262 nm at prolonged times, together with the appearance of a very small band corresponding to Pd(PPh_3_)_2_Cl_2_ at 342 nm (Figure S13). We cannot discard the possibility of a higher coordinating complex such as Pd(PPh_3_)_4_ being formed simultaneously, but the low solubility limits of both compounds, and the band overlapping of Pd(PPh_3_)_4_ and free PPh_3_ make the analysis difficult. This hypothesis, however, is supported by the fact that the hydrogenation rate is lowered when a 4:1 PPh_3_/Pd mixture is used and that higher stoichiometries do not further reduce the catalytic activity of PdCl_2_ (Figure S12). Pd(PPh_3_)_4_ has been tested as a catalyst, and it is completely inactive, which explains the observed trend. Unfortunately, so is Pd(PPh_3_)_2_Cl_2_, so whether Pd(PPh_3_)_4_ is formed cannot be confirmed, but the formation of either is indicative of the release of single Pd atoms/Pd clusters or PdCl_2_ units in the solution. Alternatively, dibenzylidene acetone (dba) was used as an additional ligand to check if Pd^0^ is present during the reaction.^32^ The catalytic results showed that the addition of dba (0.16 mol %) to the PdCl_2_-catalyzed reaction (0.04 mol % at 30 °C) decreases the hydrogenation rate of **1** (TOF_0_) from 1.1 to 0.3 s^–1^, similarly to PPh_3_ (Figure S11). Pd_2_(dba)_3_ was used as a catalyst for the reaction, and, in contrast to Pd(PPh_3_)_2_Cl_2_, the complex was active with a TOF_0_ = 1.0 s^–1^, reasonably similar to the experiment with PdCl_2_ + dba (Figures S11 and S14).^33^ Both catalysts were sonicated to increase their solubility. Additionally, classical ligands for Pd^II^ species, such as acetylacetone (acac) and acetate (OAc), were both added in 4:1 and 12:1 ligand-to-metal amounts, and the initial rates did not change with respect to those of PdCl_2_, regardless of the ligand stoichiometry. These results support the formation and catalytic role of Pd^0^ and not Pd^2+^ during the hydrogenation of **1** with PdCl_2_.^34^

### Formation and Nature of the Active Species

**Scheme 1:**
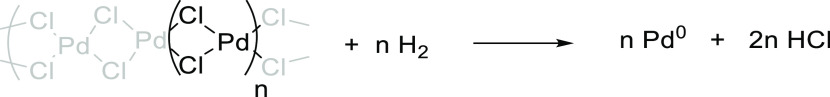
Proposed Reaction to Generate the Catalytically Active Pd^0^ Species during the PdCl_2_-Catalyzed Hydrogenation Reaction

Pd black is a very efficient catalyst, albeit not very selective at longer reaction times (Figure S15),^35^ which further supports the role of Pd^0^ in the reaction. Besides, an increase in the acidity of the reaction solution was detected by UV–vis absorption after 20 min, during which PdCl_2_ and H_2_ were put in contact (Figure S16). These results indicate the formation of a soluble acid during the reaction, which, together with the formation of Pd^0^, leads us to propose the reaction in Scheme 1 as a possible way to generate the catalytically active Pd species during the hydrogenation reaction.

**Figure 3:**
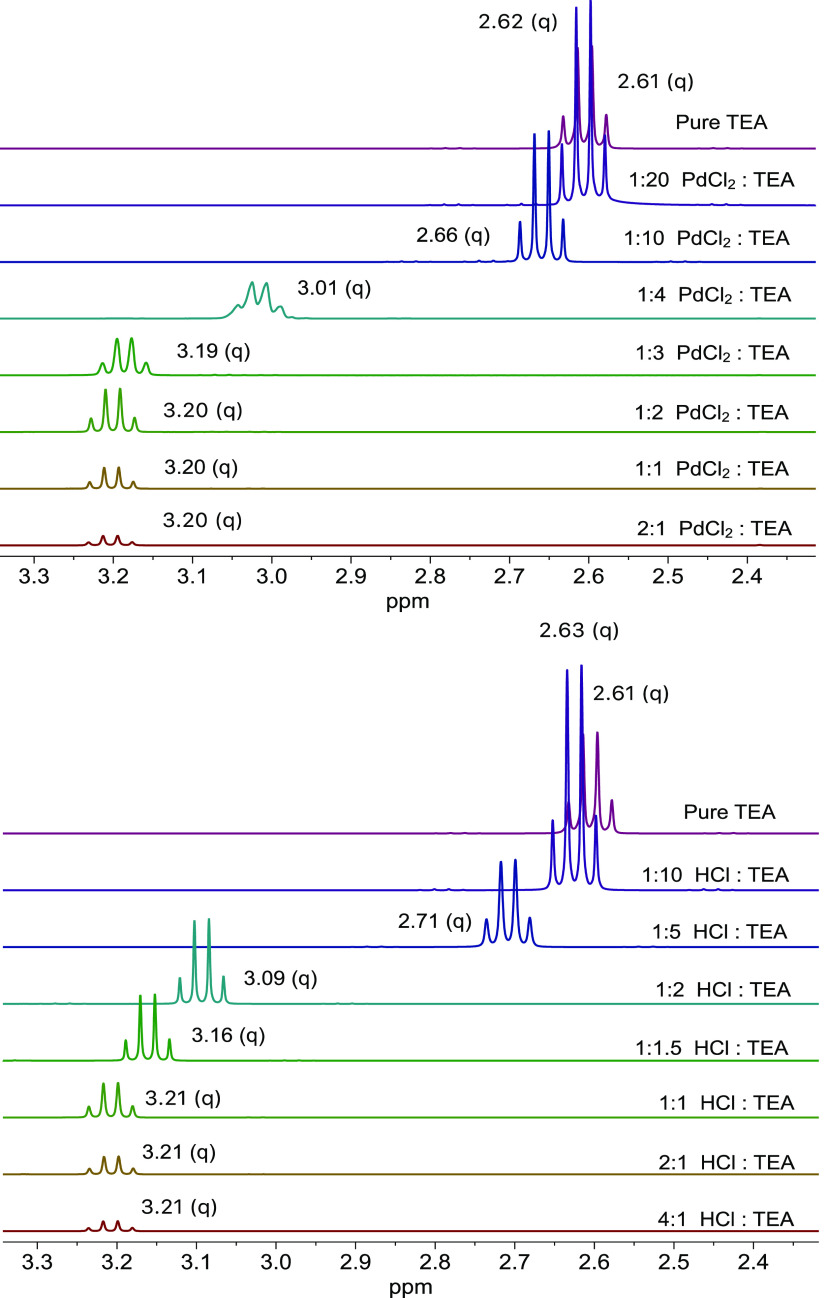
^1^H NMR of the triethylamine (TEA) methylene protons. Top: Proton shifts caused by the *in situ* formation of HCl after reduction of PdCl_2_. Bottom: Proton shifts caused by the direct addition of HCl to triethylamine solutions, at the corresponding (1:2) PdCl_2_/HCl stoichiometry. The amount of acid was kept constant throughout the experiment (0.9 mg PdCl_2_, 1 mg 37 wt % HCl in H_2_O), and the base was progressively added. The experiments were performed in D_2_O.

The reaction in Scheme 1 fits well with the need for H_2_ but not alkyne **1** to generate the active species. Thus, H_2_ reduces PdCl_2_ to Pd^0^ and generates HCl as a byproduct. Figure 3 shows ^1^H NMR studies with triethylamine (TEA), performed to precisely quantify the amount of *in situ* formed acid as the byproduct of the aforementioned reaction and to confirm the proposed mechanism for the formation of the active species.

The PdCl_2_–TEA mixtures were stirred under a H_2_ atmosphere for 1 h and measured immediately after that. The TEA methylene signal shifted from 2.61 to 3.21 ppm when an excess of acid was generated. When the base was progressively added, the signal remained unchanged until an equimolar solution was obtained. Afterward, the signal progressively shifted back to the original methylene shift of pure TEA. It is worth noting that when an excess of base is present, the protonated and unprotonated signals cannot be decoupled, which indicates a very fast acid–base equilibrium. Similar results were obtained for the methyl group proton shifts (Figure S17). When the experiment was repeated by direct addition of HCl, the same trends were observed. Thus, we can safely affirm that HCl is formed from the PdCl_2_ reduction and that Pd^0^ species are obtained and leached from the Pd^II^ catalyst.

To further assess the extent of the solution acidification, increasing Pd loadings (0.0004–10 mol %) were stirred under 5 bar of H_2_ for 30 min, after which the H_2_ was removed, and their respective pH values were measured with different probe molecules: alizarin red, 2,4-dinitrophenol, or crystal violet. According to their predicted pH values (Figure S18), all solutions were acidified in proportion to the PdCl_2_ present, thus completely reducing the Pd salt (Table S2). At this point, it is worth asserting that the acid medium did not preclude the formation of the active Pd species nor did it affect the reaction rates beyond the fact that more Pd was present in the more acidic solutions (Figure S5), a consequence of the intrinsic nature of the process. By comparing the hydrogenation reactions performed with Pd black (0.04 mol %, 30 °C, pH = 7.33, Figure S15) and PdCl_2_ (0.04 mol %, 30 °C, pH = 3.65, Figure S7), we observe that the selectivities for both reactions start falling off at ∼70% conversion, quickly dropping to a ∼30% selectivity after full alkyne conversion, which indicates that the acid medium did not improve the selectivity of the reaction. It is also worth mentioning that no cleaving of the OH group in **1** or the corresponding alkene was observed, despite being a tertiary alcohol.

To definitively discard leached discrete PdCl_2_ units as active catalytic species under the reaction conditions, soluble β-PdCl_2_ was synthesized^36^ and used as a catalyst for the hydrogenation of **1**. The structure of commercial PdCl_2_ corresponds to an insoluble polymer (where Pd and Cl atoms are disposed in a zig-zag extended configuration, with 4 Cl atoms saturating the planar 16e^–^ coordination shell of each Pd atom; crystalline γ form), but in contrast, the crystalline β form of PdCl_2_ corresponds to closed-shell hexamers, which are more soluble in organic solvents.^37^ When the more soluble, crystalline β-PdCl_2_ was used as the catalyst, an induction period and slightly lower reaction rates were observed compared to commercial γ-PdCl_2_ (Figure S19), which indicates that discrete Pd_6_Cl_12_ units from the β-phase PdCl_2_ are likely not directly involved in the hydrogenation reaction nor are they more easily reducible than the PdCl_2_ units from the commercial γ-phase. UV–vis measurements confirmed the degradation of the β-PdCl_2_ phase upon contact with H_2_ (Figure S20). The higher solubility of the β-phase results in more intense absorption bands compared to the γ-phase, although the disappearance of its bands upon mixing with H_2_ can still be observed. The X-ray diffraction (XRD) spectra obtained for the commercial γ-PdCl_2_ and the synthesized β-PdCl_2_ are in good agreement with those available in the literature (Figure S21).^37−40^ It is worth commenting here that the only available β-PdCl_2_ diffraction data in the bibliography were reported as interplanar Bragg distances, which were converted into diffraction angles for an easier comparison.^37^

**Figure 4:**
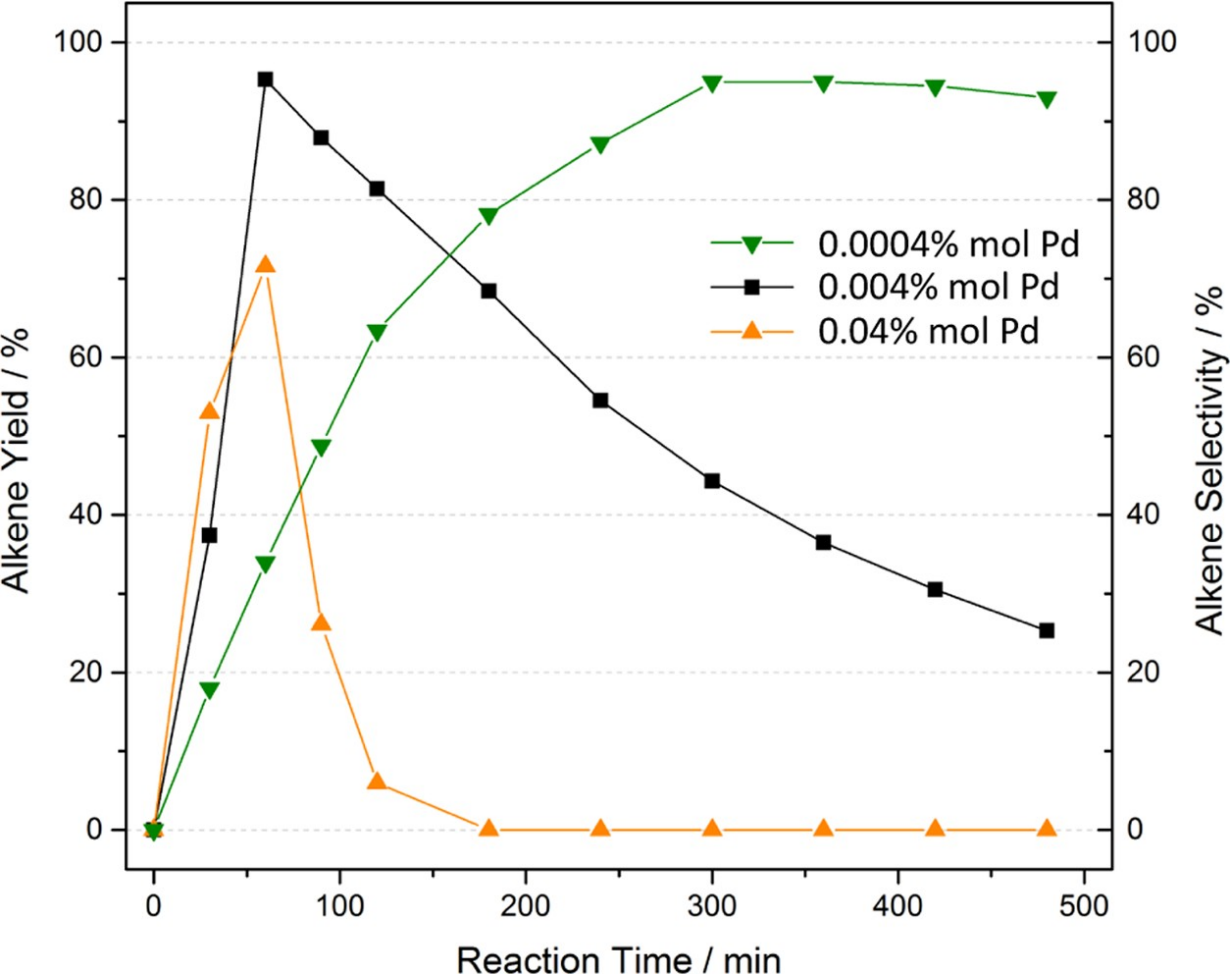
Alkene yields of the hydrogenation of **1** at 90 °C, and 5 bar of H_2_ in ethanol with PdCl_2_ at different loadings: 0.0004 mol % (green curve), 0.004 mol % (black curve), 0.04 mol % (orange curve). The reactors were previously washed with *aqua regia*. The remaining yields pertain to the corresponding alkane.

The atomicity of the Pd^0^ species in solution was then studied. The fact that >0.04 mol % PdCl_2_ does not improve the reaction rate (Figure S7) indicates that big agglomerates are plausibly not involved in the catalytic events.^41^ Besides, the reaction order for PdCl_2_ and H_2_ approximates one in both cases (Figures S5 and S22), which suggests that PdCl_2_ leaches single or very small Pd^0^ clusters to the solution to catalyze the reaction at loadings of less than 0.04 mol % Pd. In accordance, commercial Pd/C containing nanoparticles with an average particle size of 2.5 nm (in agreement with previous measurements)^42^ showed <70% selectivity for **2**, even without complete conversion of **1**. In terms of selectivity, the performance of the supported catalyst is very similar to that of the species formed at 0.04 mol % Pd, which have a similar particle size, thus in principle discarding Pd nanoparticles as the selective Pd^0^ species (Figure S15).^43^ Moreover, Figure 4 shows that the high selectivity displayed by the reaction at low Pd loadings (0.0002–0.0004 mol %) is gradually lowered at higher loadings, which points toward a nonbeneficial ripening effect.^44^ In fact, a similar effect is observed when the reaction is highly solvent-starved: the reaction does not proceed when the hydrogenation of **1** at 0.0004 mol % Pd is performed under solvent-free conditions, while the hydrogenation proceeds extremely unselectively when 20 μL of ethanol is added at the start of the reaction (where ∼110 μL of **1** was present, Figure S23), which indicates that a solvent is required for the Pd^0^ selective species to be formed and that enough solvent must be present to ensure a good dispersion of the Pd species.^45^

To shed some light on the particle size of the Pd species, UV–vis emission spectrophotometry of ethanol solutions at loadings ranging from 0.0004 to 2 mol % Pd and simultaneous high-resolution transmission electron microscopy (HR-TEM) analysis of the same solutions were performed (Figures S24 and S25).

The EM images (Figure S24) reveal a similar particle size distribution at higher loadings, 2 and 0.4%, both centered around the 50–25 nm range but with the higher loading displaying a broader distribution. At 0.04 mol %, the size distribution drastically shifts toward small nanoparticles of less than 10 nm, despite some larger aggregates still being present in the solution. At lower loadings (<0.04 mol %), although the grids were extensively loaded, no Pd species could be found. Therefore, given the high catalytic activity displayed by PdCl_2_ at parts-per-million concentrations and the inability to detect them under the microscope, it seemed clear that the species had to be sub-nanometrical.^46−48^ Simultaneously to the TEM analysis, the solutions were analyzed by emission spectrophotometry to detect Pd clusters, which are known to show different fluorescence behavior depending on their size.^49,50^ Indeed, the solutions with the lowest loadings (0.004 and 0.0004 mol % Pd) displayed a broad band from 320 to 410 nm when excited at a wavelength of 300 nm,^51^ while the other samples displayed some scattering, especially at 0.04 mol % concentration (Figure S25). Therefore, one can conclude that extremely low PdCl_2_ loadings in ethanolic solutions have been identified as very efficient and selective catalysts for the hydrogenation of **1** to **2**, a terminal aliphatic alkynol, and Pd^0^ species have been found to act as the actual catalyst of the reaction, after being formed *in situ*. Furthermore, the hydrogenation reaction proceeds less selectively in solutions in which Pd nanoparticles are formed, which highlights the selectivity of Pd clusters for the semi-hydrogenation reactions.

**Figure 5:**
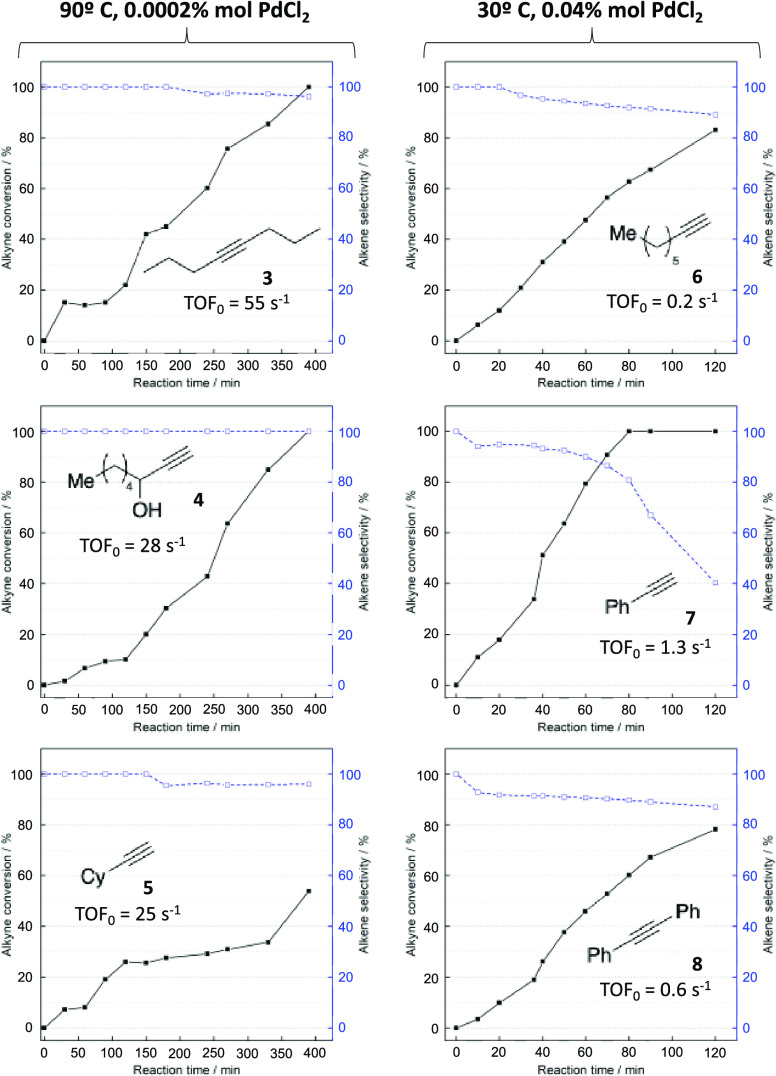
Hydrogenation of different alkynes catalyzed by the indicated amounts of PdCl_2_ in ethanol (0.5M) under 5 bar of H_2_ at 30 °C (0.04 mol % Pd) or 90 °C (0.0002 mol % Pd). The reactors were previously washed with *aqua regia*. Solid lines represent alkyne conversion, and dashed lines represent alkene selectivity; the remaining yields pertain to the corresponding alkane.

Figure 5 shows the kinetic plots for different alkynes. 4-Octyne **3**, 1-octyn-3-ol **4**, and cyclohexylacetylene **5** are hydrogenated by 0.0002 mol % PdCl_2_ catalyst at 90 °C with TOF_0_’s > 25 s^–1^ and selectivity >96% to the corresponding alkene (>95% *cis* for **3**). However, 1-octyne **6**, phenylacetylene **7**, and diphenylacetylene **8** require an increase in the amount of PdCl_2_ to 0.04 mol % in order for the reactions to proceed. The selectivity toward their corresponding alkenes is lower (>80%, >90% *cis* for **8**), which was expected at this loading (Figure 4). It is worth mentioning that there is evidence of diphenylacetylene hydrogenation with PdCl_2_ in the bibliography, although the process was not selective to the alkene.^52^ The authors were using high PdCl_2_ loadings (0.94 mM), and according to our results, reactions performed with Pd concentrations higher than 0.1 mM (0.04 mol %, under our conditions) unselectively catalyze the hydrogenation reactions. Higher loadings enable milder temperatures (30 °C) but lower the intrinsic activities of the catalyst (TOF_0_’s ∼ 1 s^–1^).

At these reaction conditions, however, most of the Pd can be recovered after reaction by simple centrifugation. This recovered Pd can be reused for the next batch, keeping most of the catalytic activity even after being exposed to ambient conditions (Figure S26). In accordance with the above results, this recovered Pd must be in a reduced oxidation state. Representative gas chromatography (GC) and gas chromatography-mass spectrometry (GC–MS) spectra of the reactions are included in the Supporting Information (Figure S27).

The fact that extremely low amounts of PdCl_2_, a primary salt in the Pd production chain, are able to selectively catalyze the semi-hydrogenation reaction with such a huge efficiency beyond the reactive differences found for different alkynes makes this catalytic system promising for the development of industrial hydrogenation reactions.^53,54^ Not only that, the catalytically active, ligand-free Pd^0^ atoms are generated *in situ* by the sole action of H_2_, so they could have general use for other chemical transformations after removing or preserving the H_2_ atmosphere.

## Conclusions

The semi-hydrogenation of 3-methyl-1-pentyn-3-ol **1** was satisfactorily performed (*X* = 100%, *S* ∼ 95%) with parts-per-million amounts of Pd (0.0002 mol %). The active Pd species were found to be Pd^0^, which were formed *in situ* by the reductive H_2_ atmosphere. Their size was a key factor in maintaining a high selectivity toward the alkene formation.^55^ At these loadings (0.0002–0.0004 mol % Pd), where large Pd aggregates were not found, the selectivity was maintained even after full conversion of the alkene, which underlines the detrimental effect of Pd NPs on the selectivity of semi-hydrogenation reactions. Despite the fact that the performance seems to be very substrate-dependent, these Pd-starved solutions show promise as a great framework in which more sustainable selective hydrogenations can be performed through a more efficient use of a scarce noble metal. The ability of isolated Pd atoms/Pd clusters to catalyze the selective semi-hydrogenation is a current subject of study nowadays,^56−58^ an endeavor toward the simplification of the more traditional systems, where the Pd species were isolated through lead poisoning, such as the Lindlar catalyst,^59^ or by more sophisticated adsorbates found in contemporary systems.^60^

## Experimental Methods

### Materials

All chemicals and Pd salts used were of reagent grade quality. Except for the β-PdCl_2_ phase, all were purchased from commercial sources and used as received.

### Synthesis of the β-PdCl_2_ Phase

Pd(OAc)_2_ (0.5 mmol) was placed in a 50 mL round-bottom flask, into which 25 mL of glacial acetic acid was poured. After stirring for 30 min to fully dissolve the Pd salt, 1 mmol of HCl was added dropwise into the flask while stirring. The HCl was previously diluted in 1 mL of acetic acid. Upon addition, the solution became turbid and part of the Pd started precipitating. While the β-phase is more soluble than the commercial phase, it is much less soluble than Pd(OAc)_2_ and it rapidly saturates and precipitates out. After 1 h, the stirring was stopped, and the formed PdCl_2_ was separated by centrifugation, washed with deionized water, and dried overnight under vacuum at 80 °C. The final yield of β-PdCl_2_ after washing and drying was approximately 90% in weight.

### Physical Techniques

The metal content of the samples was determined by inductively coupled plasma-optical emission spectroscopy (ICP–OES) (Thermo Scientific ICAP Pro). Solids were disaggregated in aqua regia and later diluted in water before analysis. Attenuated total reflection infrared spectroscopy, performed in a JASCO FT/IR-4000, was employed to record the IR spectra of the solutions after the reaction (400–4000 cm^–1^), by dropping a small sample of the solution on the ATR crystal. GC and GC–MS chromatography were performed in gas chromatographs with 25 m capillary columns filled with 1 or 5 wt % phenylsilicone (Shimadzu GC-2025, Agilent GC 6890N coupled with Agilent MS-5973). ^1^H NMR spectra were recorded at room temperature on a 400 MHz spectrometer (Bruker Ascend 400). Absorption spectra were recorded on an Agilent Cary 60 UV–Vis spectrophotometer in 1 cm wide cuvettes with a xenon source lamp. Fluorescence emission spectra were recorded on a FLS1000 photoluminescence spectrometer from 320 to 650 nm, with a xenon source lamp at an excitation wavelength of 300 nm. X-ray diffraction spectra of the different phases of PdCl_2_ were recorded in a CubiX PRO (PAN Analytical) spectrometer with a Cu K(α) radiation source at 1.5406 Å wavelength.

### Electron Microscopy Characterization

The images of the catalyst were obtained on a JEM-F2100 operated at 200 kV in dark field scanning transmission electron microscopy (DF-STEM mode). The solutions at different Pd loadings were accordingly loaded onto the grids to compensate for the lack of sample in each one.

### Semi-Hydrogenation Reactions

All of the batch reactions were performed in a 20 mL autoclave reactor with a stirring magnet. The reactions were conducted at 30–90 °C and stirred at 450 rpm in an H_2_ pressurized atmosphere of 5 bar unless otherwise stated. The yields were obtained by gas chromatography and gas chromatography-mass spectrometry. The products were characterized by NMR and were compared with the available literature.

## Supporting Information Available

Additional experimental data, including kinetics, characterization of the catalyst and products, and comparison of the catalytic system with the literature precedents, among others, are provided in Figures S1–S27 and Tables S1 and S2 (PDF)
