# Supplementary material for: Parts-Per-Million of Soluble Pd0 Catalyze the Semi-Hydrogenation Reaction of Alkynes to Alkenes
Source: J Org Chem. 2022 May 18;88(1):18–26. doi: 10.1021/acs.joc.2c00616 (PMC9830639; doi:10.1021/acs.joc.2c00616)
Supplement: Supplementary file 1 — jo2c00616_si_001.pdf [file jo2c00616_si_001.pdf]

SUPPORTING INFORMATION

Parts-per-million of soluble Pd^0 catalyze the semi-hydrogenation reaction of alkynes to alkenes

Jordi Ballesteros-Soberanas,[a,‡] Jose A. Carrasco[a,‡] and Antonio Leyva-Pérez.[a,*]

[a] Instituto de Tecnología Química. Universitat Politècnica de València-Consejo Superior de Investigaciones Científicas. Avda. de los Naranjos s/n, 46022, Valencia, Spain.

‡ These authors contributed equally.

* Corresponding author, e-mail: anleyva@itq.upv.es.

Table of contents

Supporting Figures (Figures S1 - S27) p. S2-S33

Supporting Tables (Table S1-S2) p. S34-S35

References p. S36

Supporting Figures

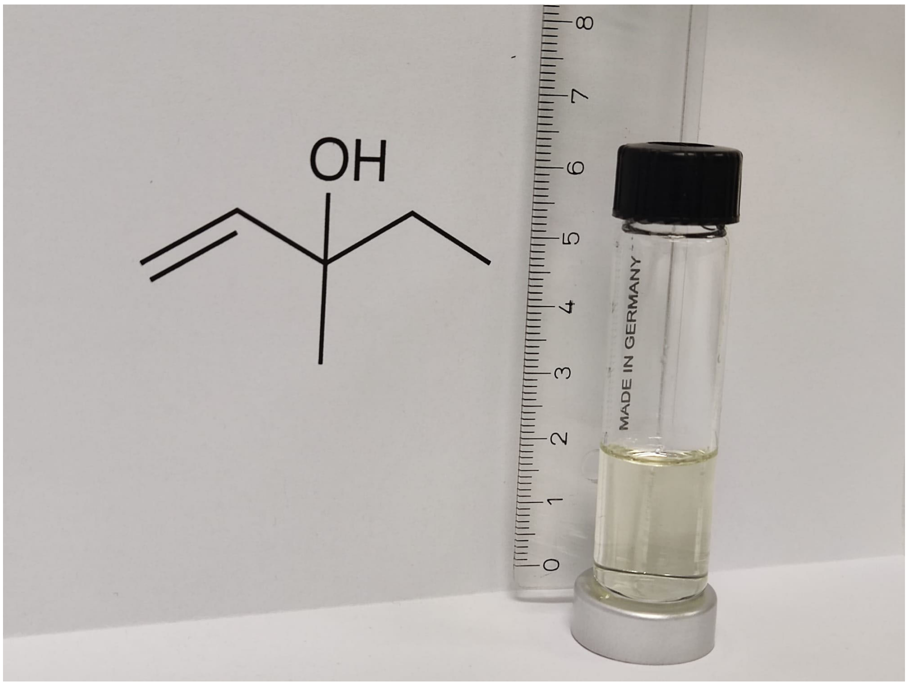

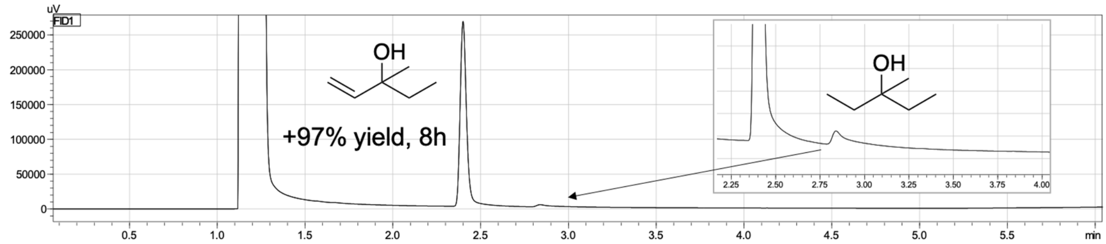

Figure S1. Product of the hydrogenation reaction with 3 g of **1** to **2** in ethanol (0.5M) under 8 bars of H_2 at 90 ºC, with $PdCl_2$ (0.0004 mol% Pd). The image shows the reaction product (97% of **2**, 3% of the corresponding alkane, in weight, diluted in approximately 20 % of ethanol to prevent polymerization of **2**). The mixture can be safely stored at -20 ºC for days with no signs of degradation. The GC spectra is included for reference. See Figure S25 for GC-MS spectra.

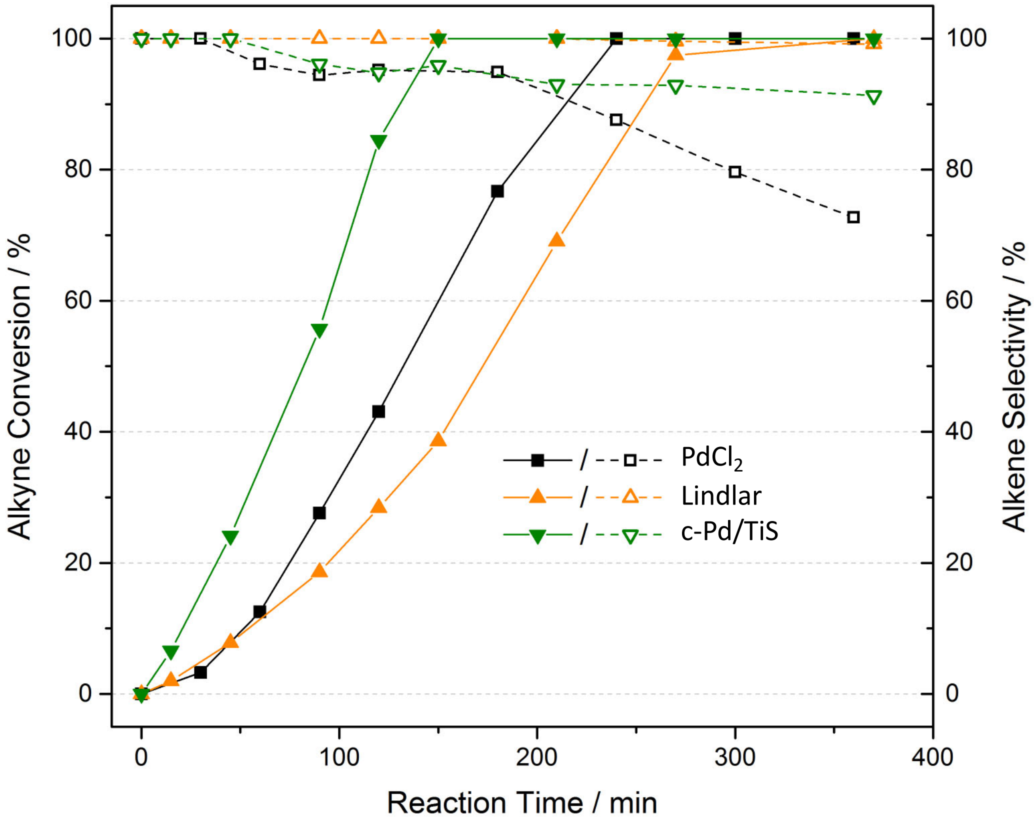

Figure S2. Kinetic plots for the hydrogenation of **1** to **2** with $PdCl_2$ (0.004 mol% Pd), Lindlar and c-Pd/TiS catalysts (0.04 mol%), in ethanol (0.5M) under 5 bars of H_2 at 30 ºC. The reactors were previously washed with *aqua regia.* Solid lines represent alkyne conversion and dashed lines represent alkene selectivity, the balance is the corresponding alkane.

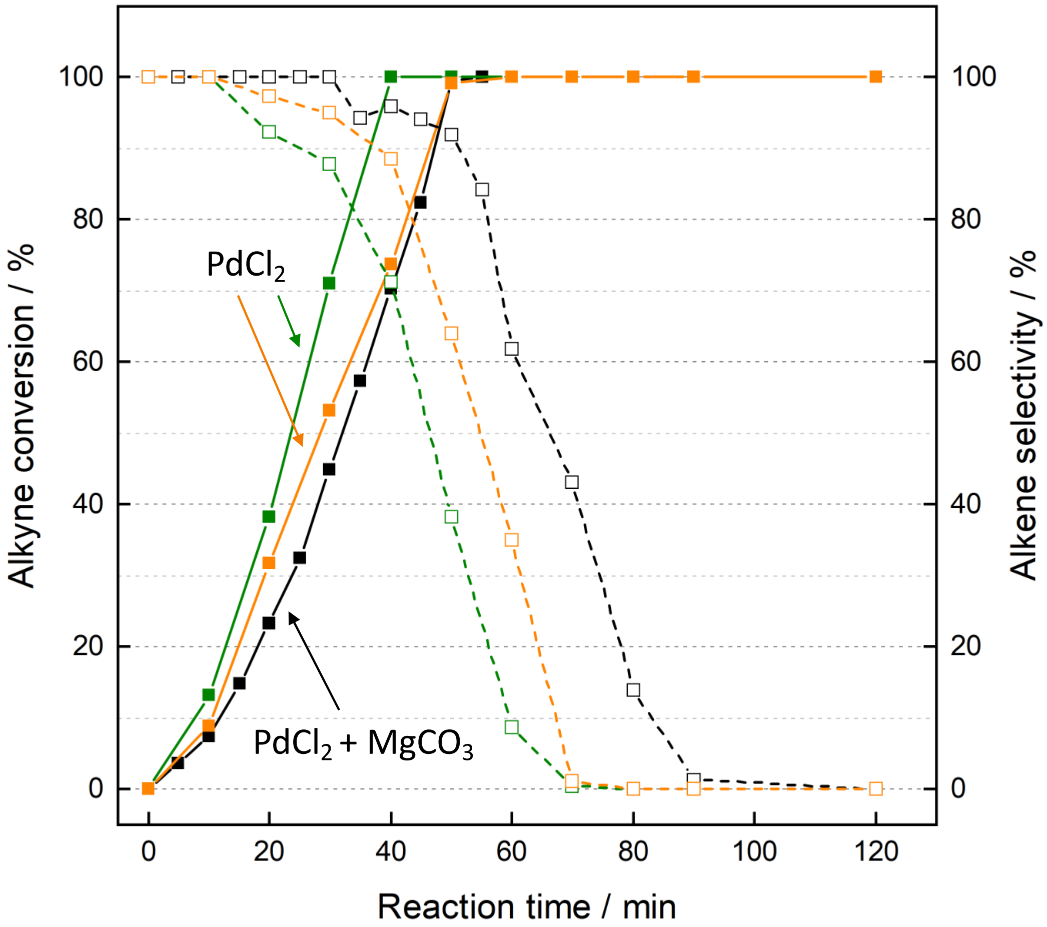

Figure S3. Kinetic plots for the hydrogenation of **1** to **2** with 0.04 mol% of $PdCl_2$ (green and orange lines, two different batches) or $PdCl_2$ with $MgCO_3$ (black lines) in ethanol (0.5M) under 5 bars of H_2 at 30 ºC. Solid lines represent alkyne conversion and dashed lines represent alkene selectivity, the balance is the corresponding alkane.

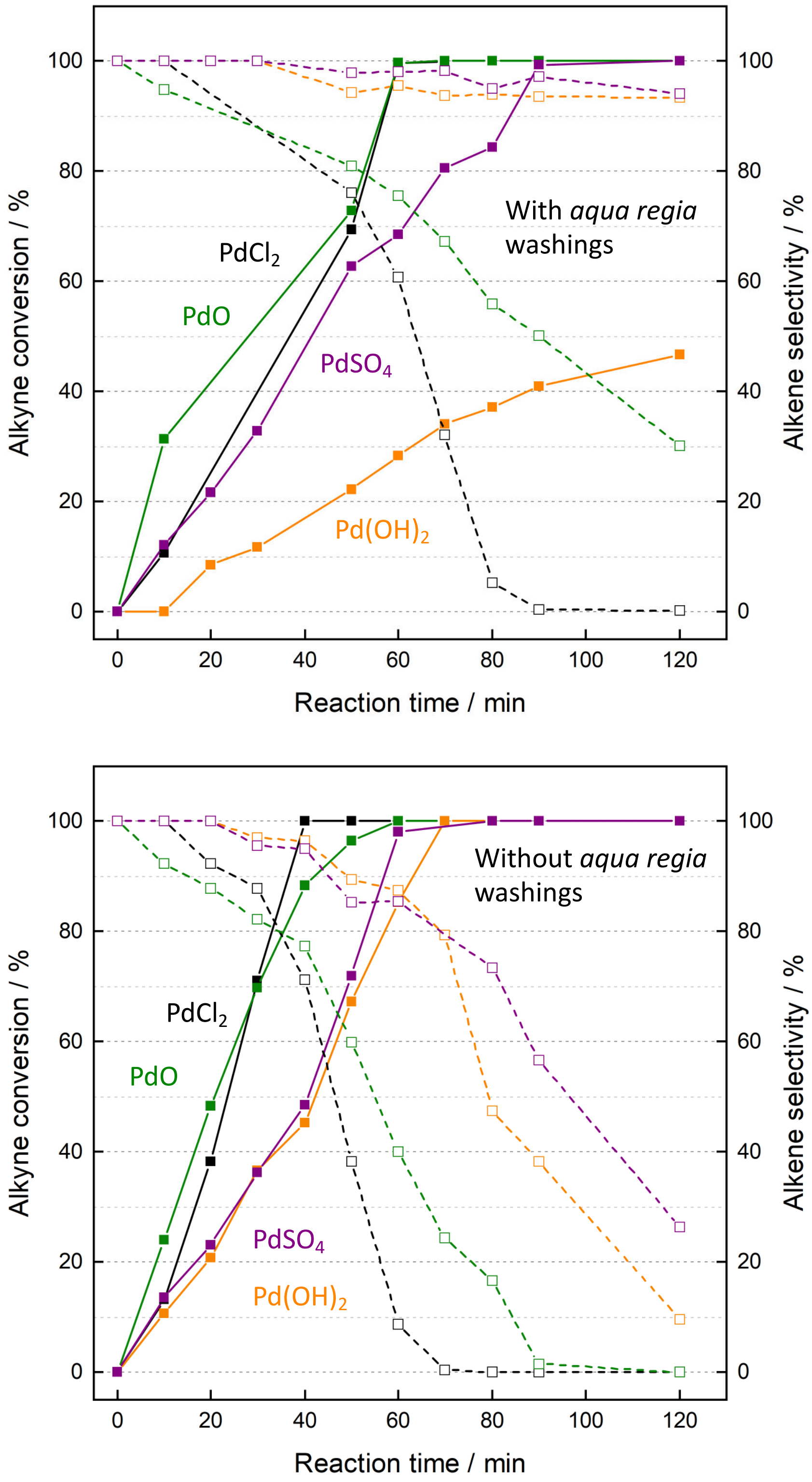

Figure S4. Kinetic plots for the hydrogenation of **1** to **2** with 0.04 mol% of PdO (green lines), $Pd(OH)_2$ (orange lines), $PdSO_4$ (magenta lines) or $PdCl_2$ (black lines), in ethanol (0.5M) under 5 bars of H_2 at 30 ºC, after washings of the reactor with *aqua regia* (top) or without *aqua regia* (bottom). Solid lines represent alkyne conversion and dashed lines represent alkene selectivity, the balance is the corresponding alkane.

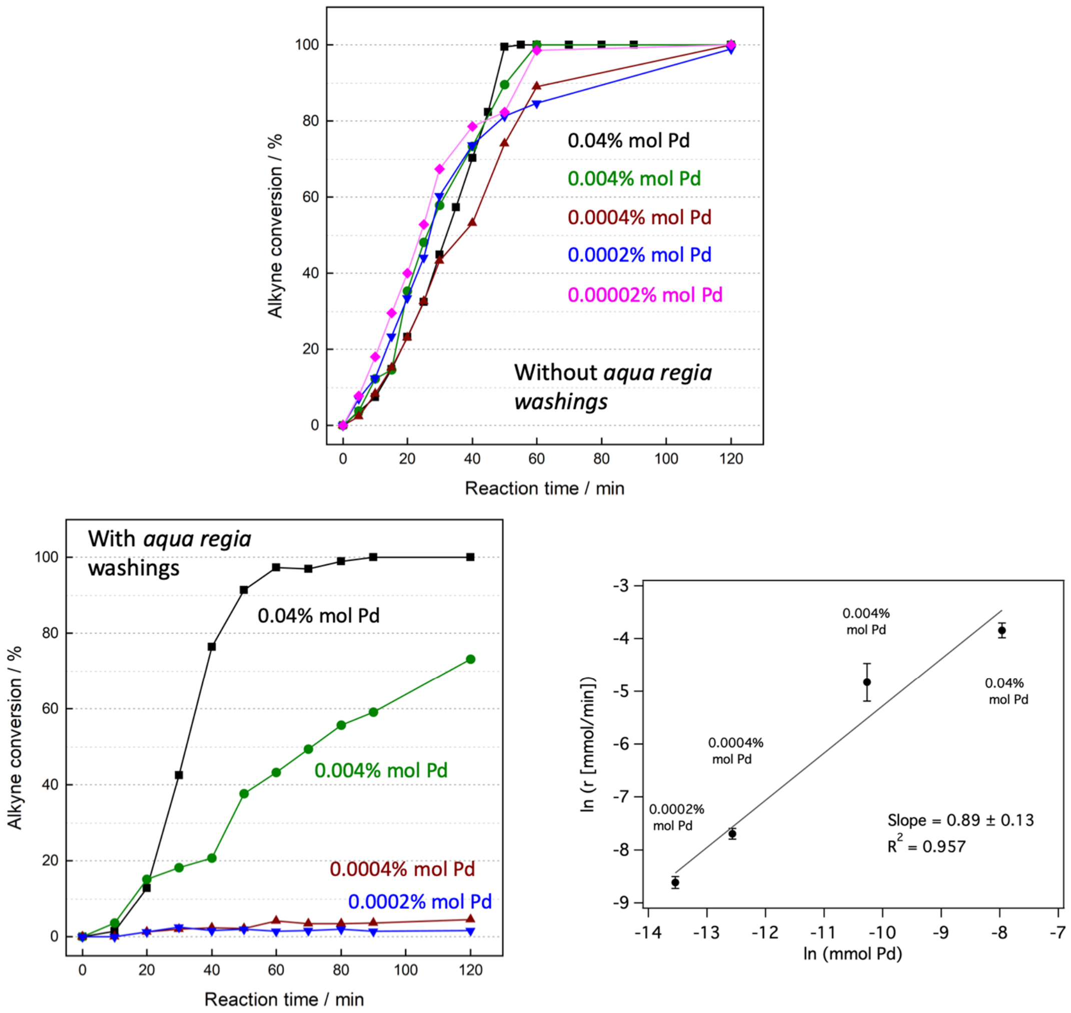

Figure S5. Top: Kinetic plots for the hydrogenation of **1** to **2** with different amounts of $PdCl_2$ on $MgCO_3$ in ethanol (0.5M) under 5 bars of H_2 at 30 ºC. Bottom left: the same reactions after washing with *aqua regia* form batch to batch. For amounts of Pd >0.1 mol%, the *aqua regia* washings have no effect in rate variation. Bottom right: Estimation of the reaction order for Pd.

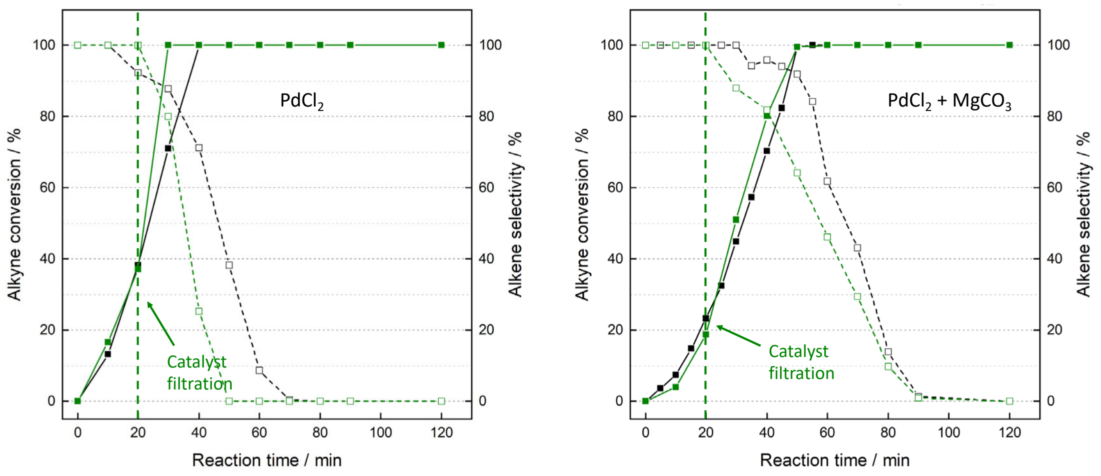

Figure S6. Kinetic plots for the hydrogenation of **1** to **2** with 0.1 mol% of $PdCl_2$ (left) or $PdCl_2$ on $MgCO_3$ (right) in ethanol (0.5M) under 5 bars of H_2 at 30 ºC, after filtering the solid catalyst at 20 min reaction time (green lines), alongside the control runs, which remained unaltered (black lines). The reactors were previously washed with *aqua regia.* Solid lines represent alkyne conversion and dashed lines represent alkene selectivity, the balance is the corresponding alkane.

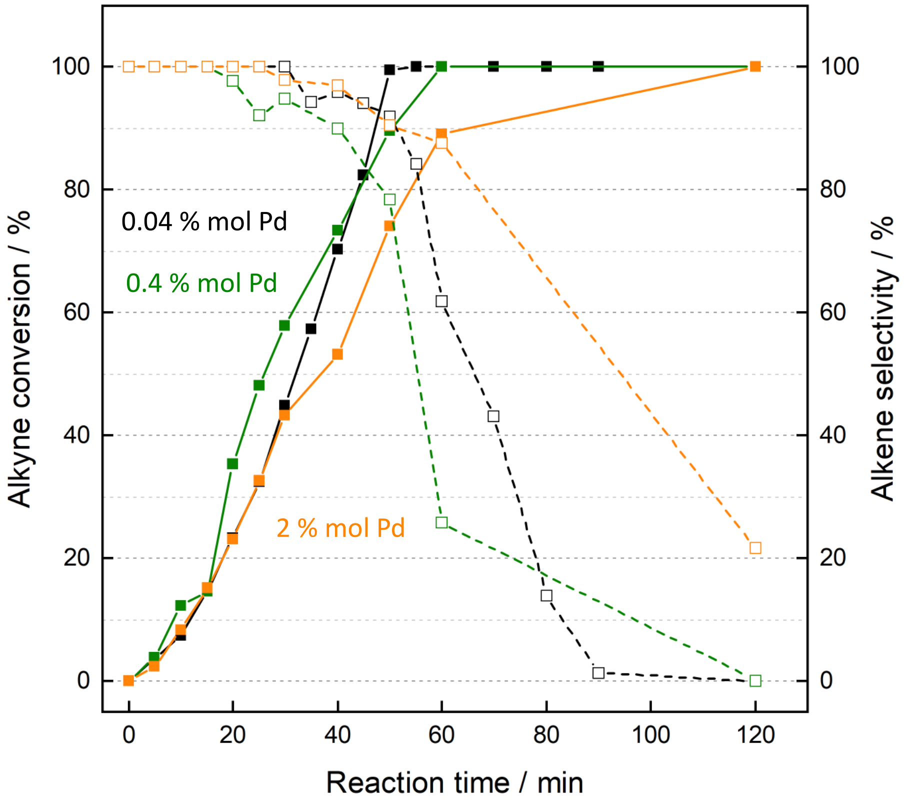

Figure S7. Kinetic plot for the hydrogenation of **1** to **2** with 0.04 - 2 mol% of $PdCl_2$ in EtOH (0.5M) under 5 bars of H_2 at 30 ºC. Solid lines represent alkyne conversion and dashed lines represent alkene selectivity, the balance is the corresponding alkane.

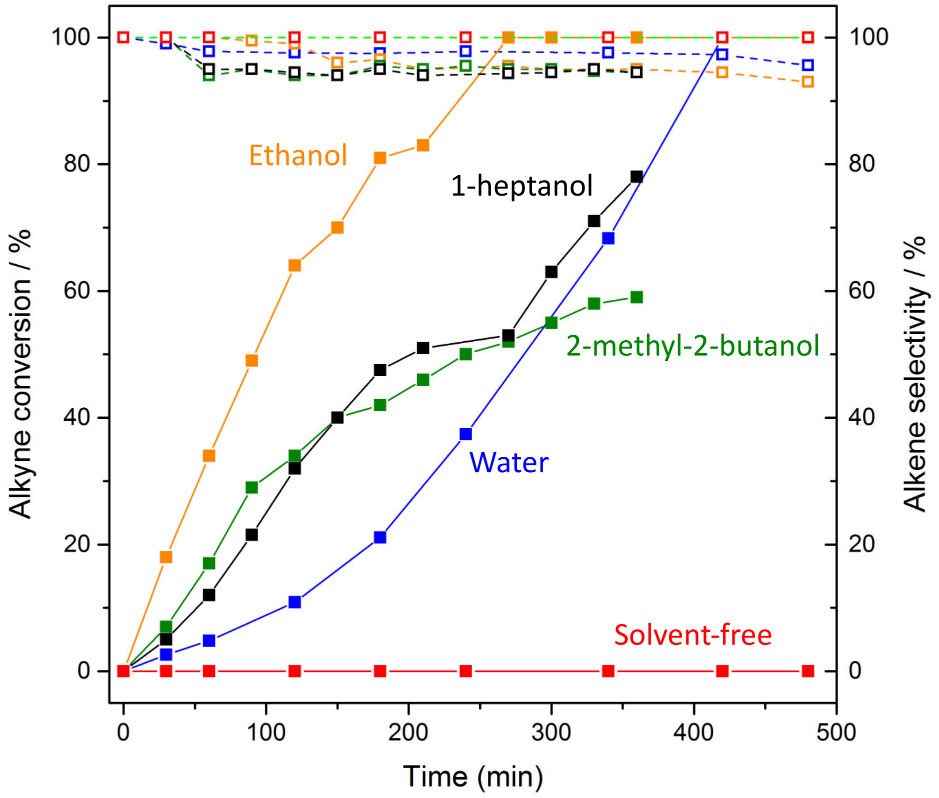

Figure S8. Kinetic plots for the hydrogenation of **1** to **2** with 0.0002 mol% of $PdCl_2$ under 5 bars of H_2 at 90 ºC, using a 0.5M solution of either EtOH (orange lines), 1-heptanol (black lines), 2-methyl-2-butanol (green lines), water (blue line) or solventless conditions (red lines). The reactors were previously washed with *aqua regia.* Solid lines represent alkyne conversion and dashed lines represent alkene selectivity, the balance is the corresponding alkane.

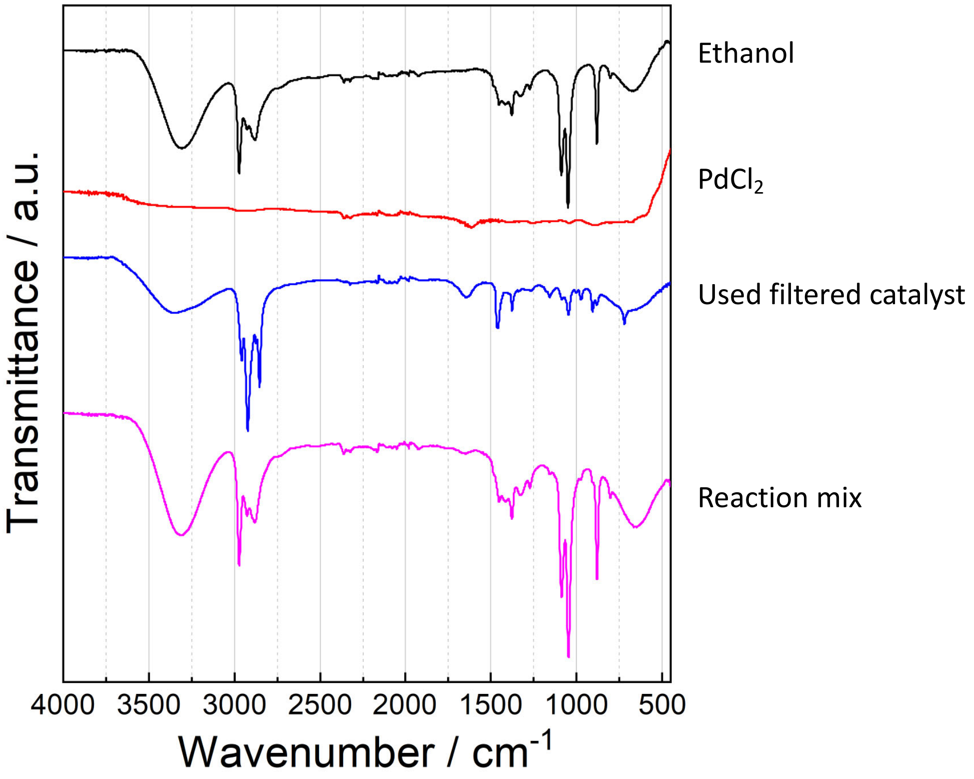

Figure S9. Fourier transformed infrared spectroscopy (FT-IR) spectra of the reaction mixture after hydrogenation of **1** to **2** with 0.1 mol% of $PdCl_2$ in EtOH.

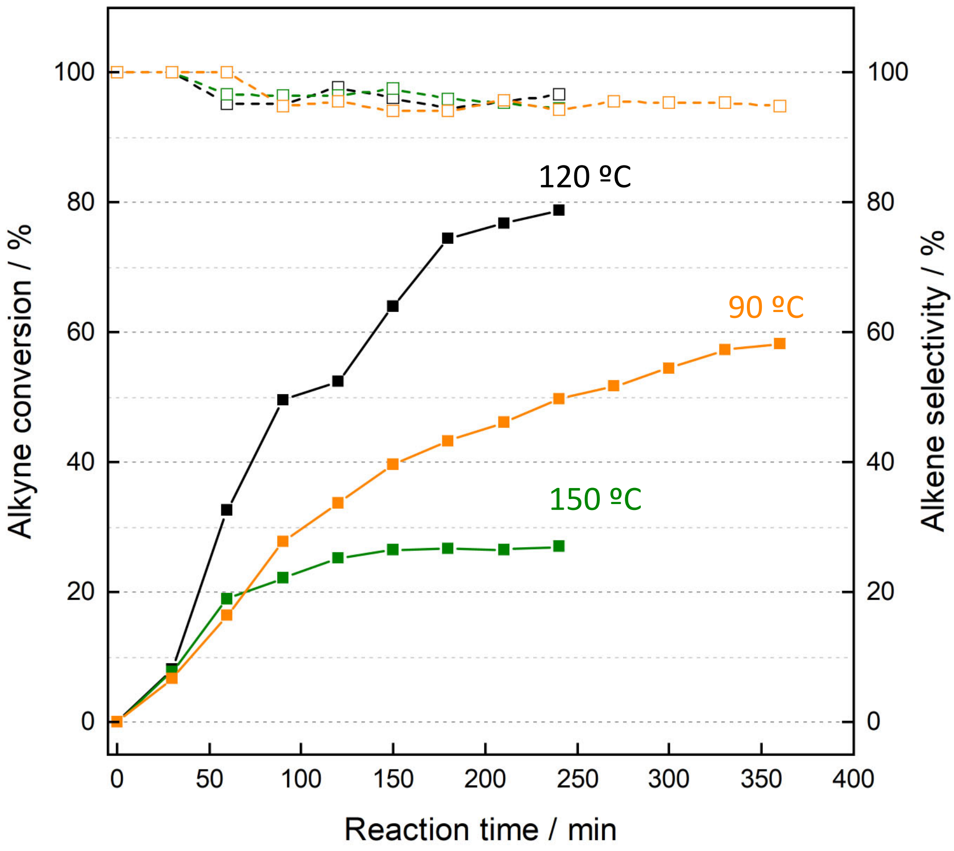

Figure S10. Kinetic plots for the hydrogenation of **1** to **2** with 0.0002 mol% of $PdCl_2$ at different reaction temperatures under 5 bars of H_2, using a 0.5M solution of 2-methyl-2-butanol. The reactors were previously washed with *aqua regia.* Solid lines represent alkyne conversion and dashed lines represent alkene selectivity, the balance is the corresponding alkane.

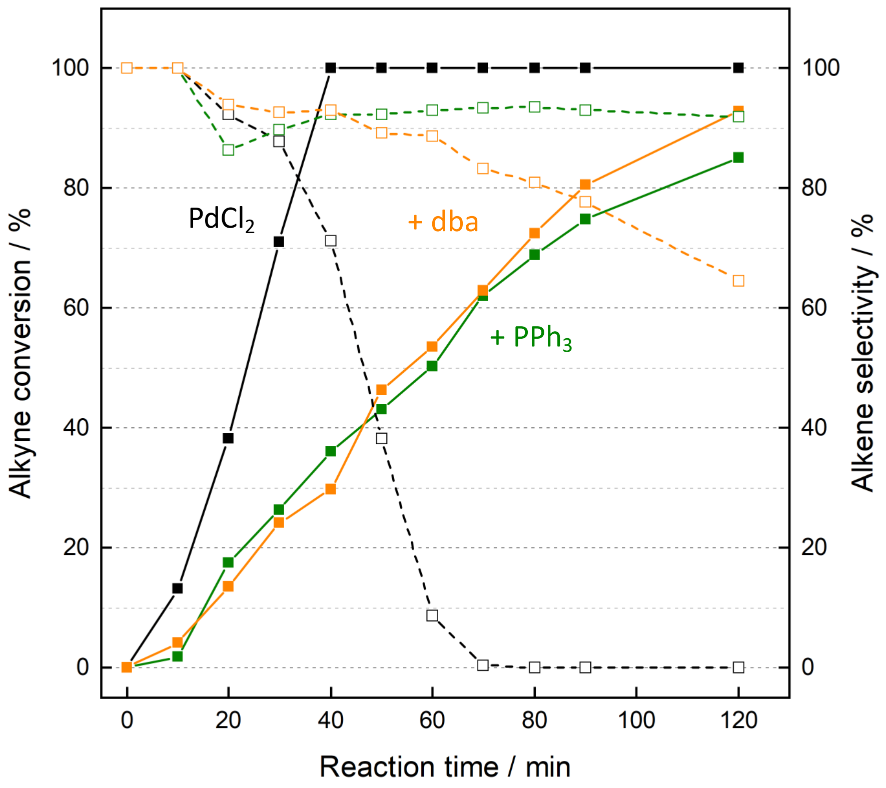

Figure S11. Kinetic plots for the hydrogenation of **1** to **2** with 0.04 mol% of $PdCl_2$ in EtOH (0.5M) under 5 bars of H_2 at 30 ºC (black lines), the 0.16 mol% PPh_3 poisoned run (green lines) and the 0.16 mol% dibenzylidene acetone poisoned run (dba, orange lines). The reactors were previously washed with *aqua regia*. Solid lines represent alkyne conversion and dashed lines represent alkene selectivity, the balance is the corresponding alkane.

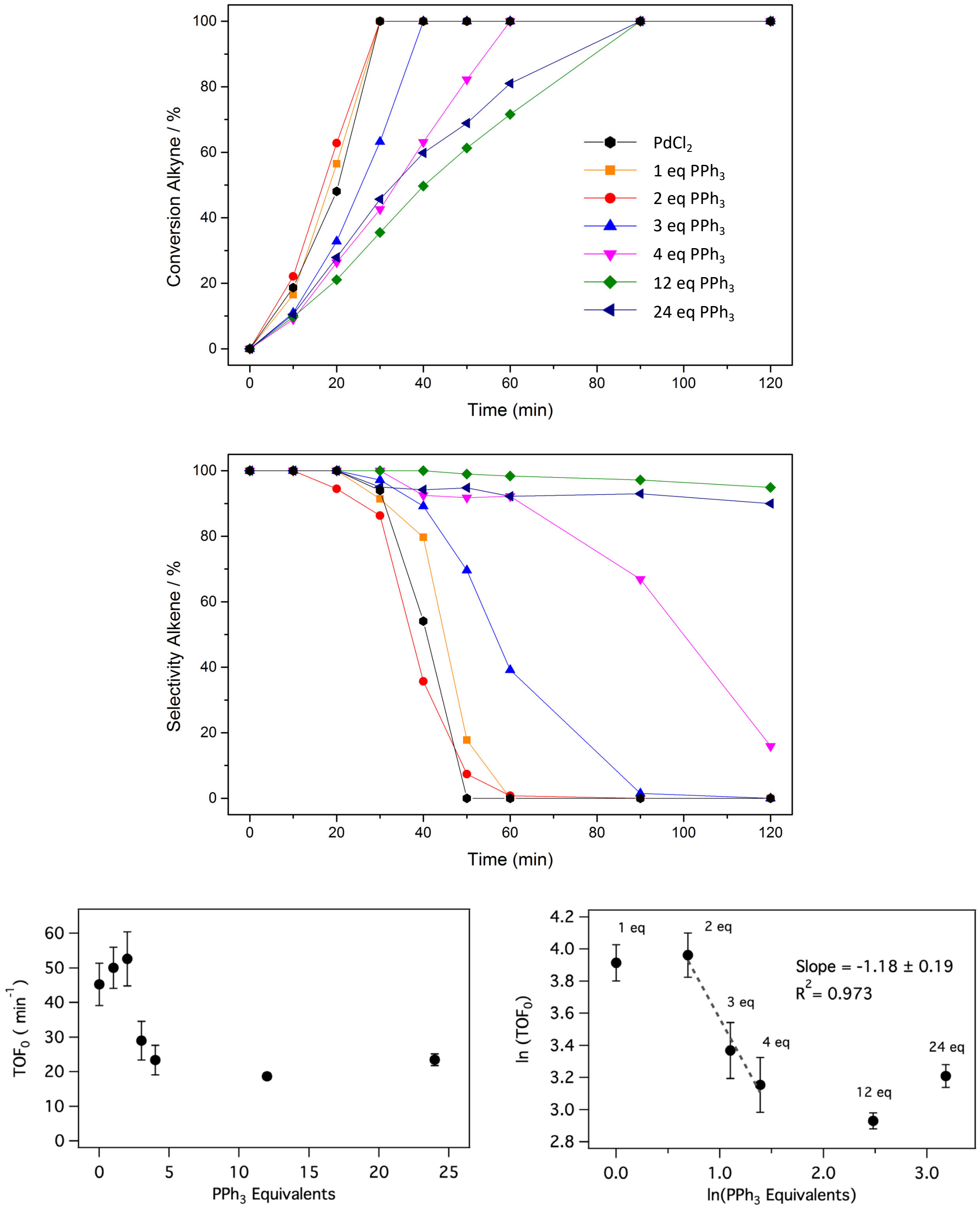

Figure S12. Kinetic plots for the hydrogenation of **1** to **2** with 0.04 mol% of $PdCl_2$ in EtOH (0.25M) under 5 bars of H_2 at 30 ºC (black lines), and the 0.04 mol% (orange), 0.08 mol% (red), 0.12 mol% (blue), 0.16 mol% (pink), 0.48 mol% (green) and 0.96 mol% of PPh_3. The reactors were previously washed with *aqua regia.* Top: conversion plots; middle: selectivity plots to alkene **2**; bottom, left: initial rates *vs*. PPh_3 equivalents; bottom, right: PPh_3 apparent reaction order in the linear regime.

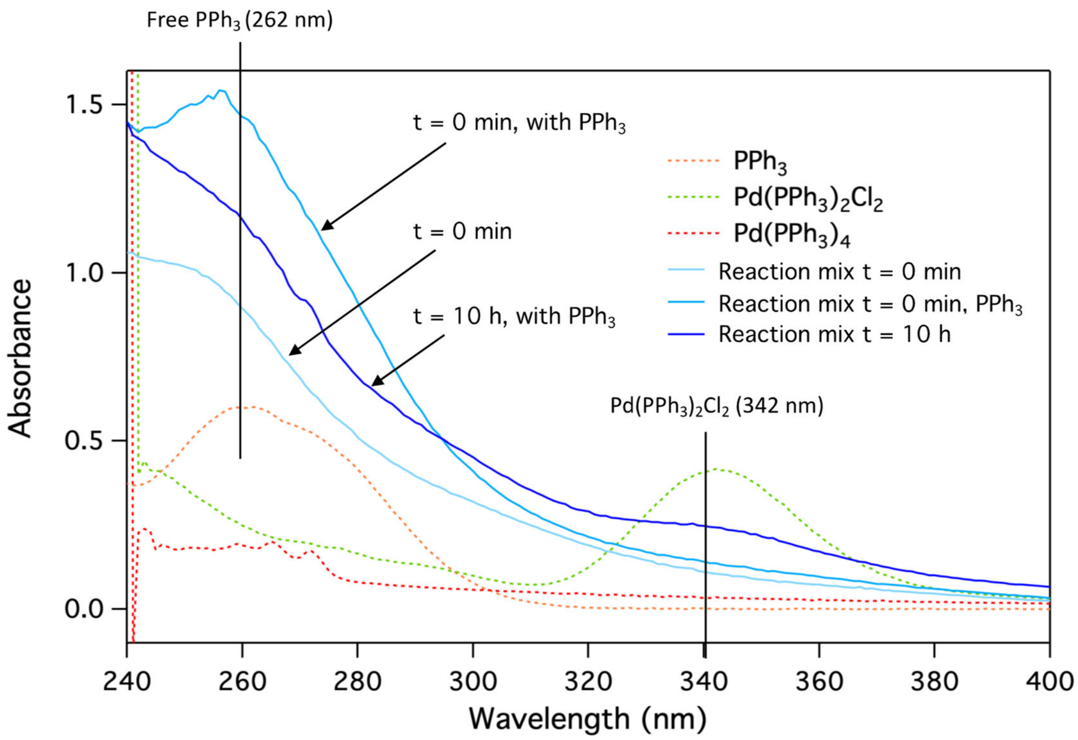

Figure S13. Ultraviolet-visible (UV-vis) absorption spectra for the hydrogenation of **1** to **2** with 0.04 mol% of $PdCl_2$ and 0.16 mol% of PPh_3, in EtOH (0.5M) under 5 bars of H_2 at 30 ºC. The reactors were previously washed with *aqua regia.* Solid lines represent alkyne conversion and dashed lines represent alkene selectivity, the balance is the corresponding alkane.

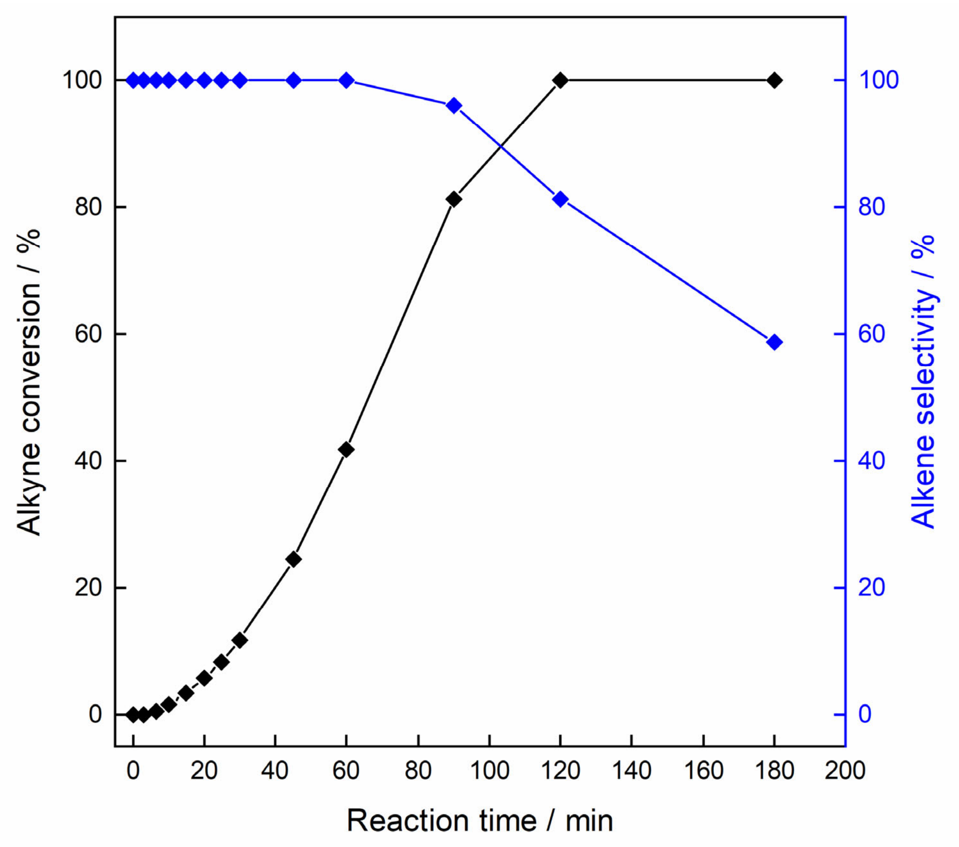

Figure S14. Kinetic plots for the hydrogenation of **1** to **2** with 0.1 mol% of $Pd_2(dba)_3$ in EtOH (0.5M) under 5 bars of H_2 at 30 ºC. Solid lines represent alkyne conversion and dashed lines represent alkene selectivity, the balance is the corresponding alkane.

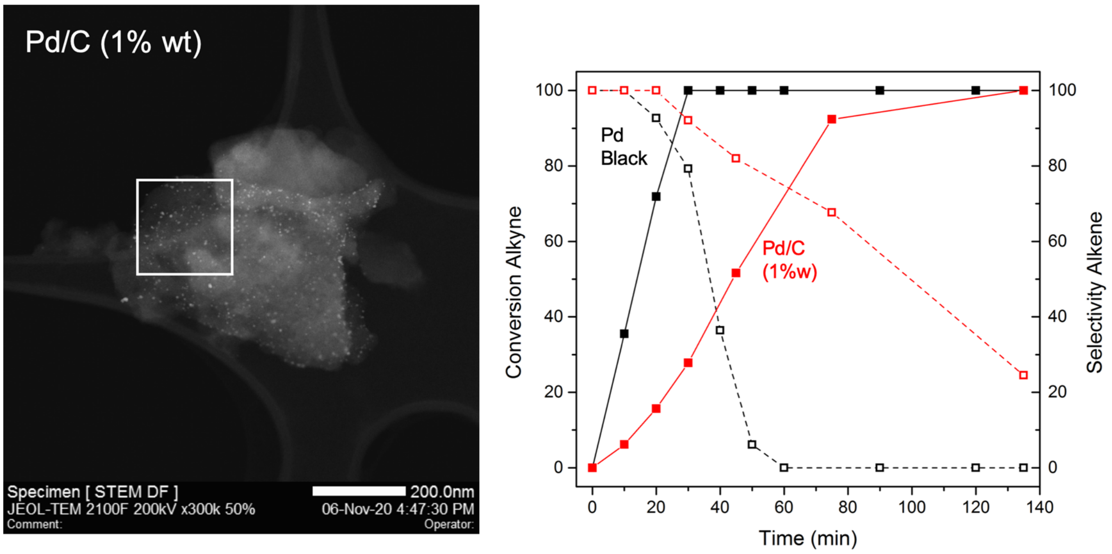

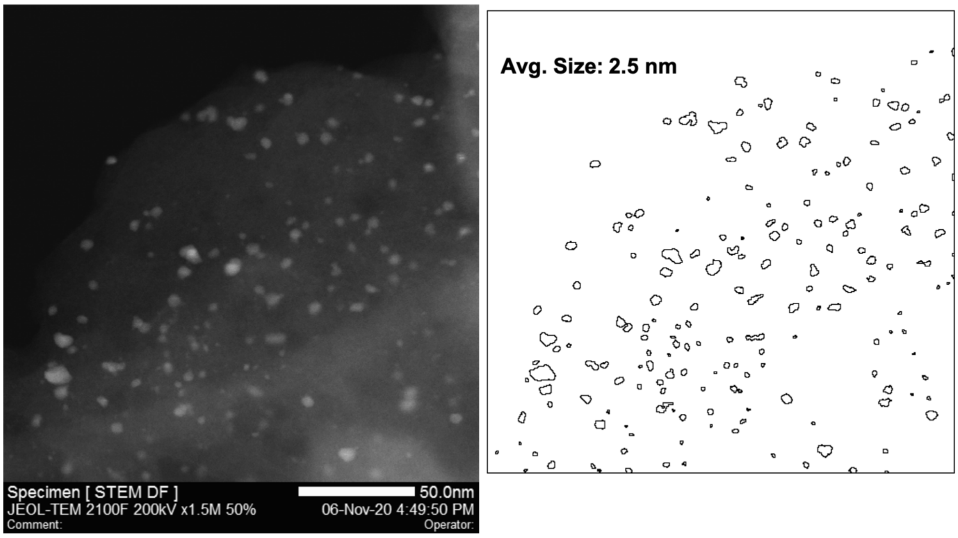

Size (nm)	Counts
8	1
7	1
6	4
5	2
4	33
3	45
2	54
1	44
	184

Figure S15. Top right: kinetic plots for the hydrogenation of **1** to **2** (0.5M in ethanol). Black lines: 0.04 mol% of Pd, palladium black, 30 ºC, 5 bar H_2. Red lines: 0.004 mol% of Pd, palladium on carbon (nanoparticles, 1 wt%), 30 ºC, 3 bar H_2. The solid lines represent alkyne conversion and the dashed lines represent alkene selectivity, the balance is the corresponding alkane. Left, top and bottom: Dark Field STEM images of the Pd/C (1%w) catalyst. Bottom right: particle mapping and particle size analysis. The images were processed to enhance contrast and reduce undesired glares, previous to the computer assisted particle outlining. The identification, mapping, and particle size estimation of the particles was performed in Image-J (Fiji package)[S1].

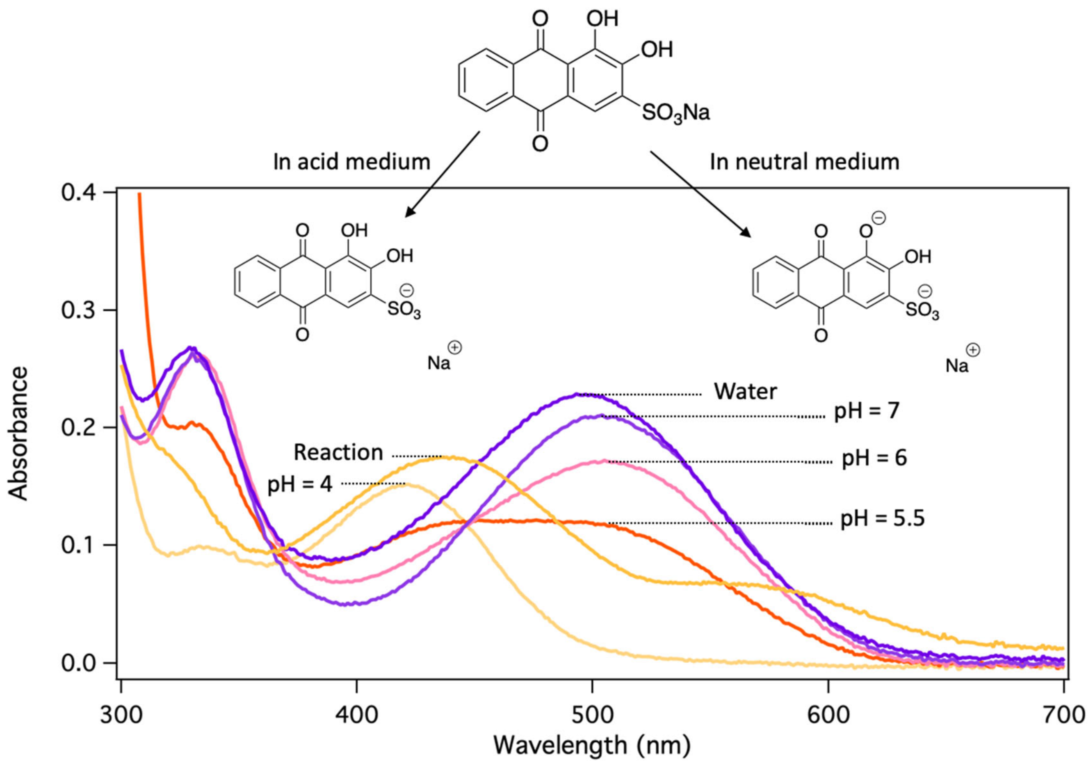

Figure S16. UV-vis absorption spectrophotometry of the reaction mixture after 20 minutes during the hydrogenation of **1** to **2** with 0.04 mol% of $PdCl_2$ in EtOH (0.5M) under 5 bars of H_2 at 30 ºC. The reaction was placed in the UV-Vis cuvette and alizarin red was added to the reaction mixture (dark yellow line). The other curves were obtained for pH calibration purposes, by mixing alizarin red with different aqueous pH buffers. The colors of the UV-vis curves approximate the actual colors of the solutions.

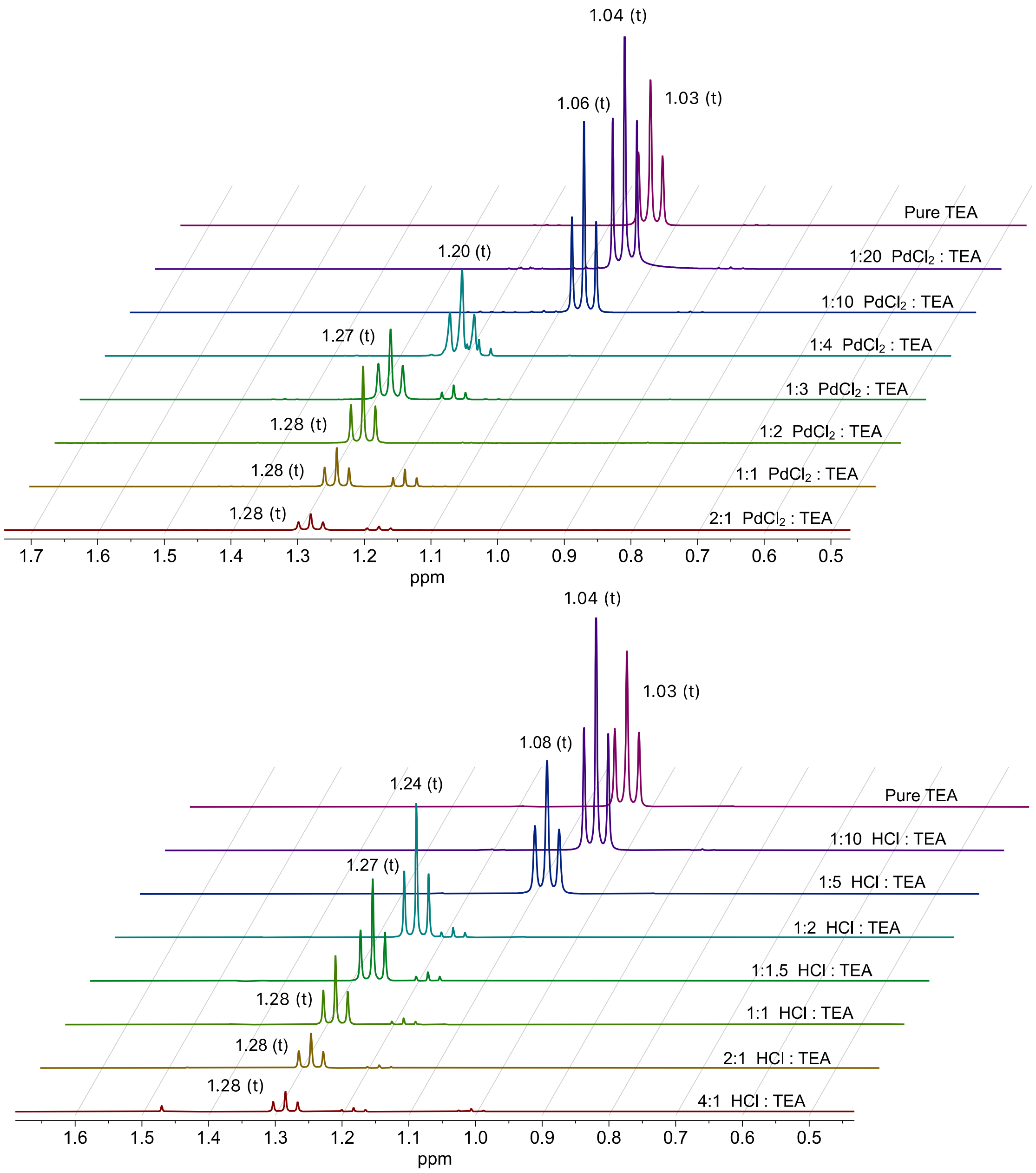

Figure S17. ^{1}H-NMR of the triethylamine methyl protons. Top: proton shifts caused by the in-situ formation of HCl after reduction of $PdCl_2$. Bottom: proton shifts caused by the direct addition of HCl to triethylamine solutions, at the corresponding (1:2) $PdCl_2$:HCl stoichiometry. The amount of acid was kept constant throughout the experiment (0.9 mg $PdCl_2$, 1 mg 37%w HCl in H_2O) and the base was progressively added. The experiments were performed in D_2O.

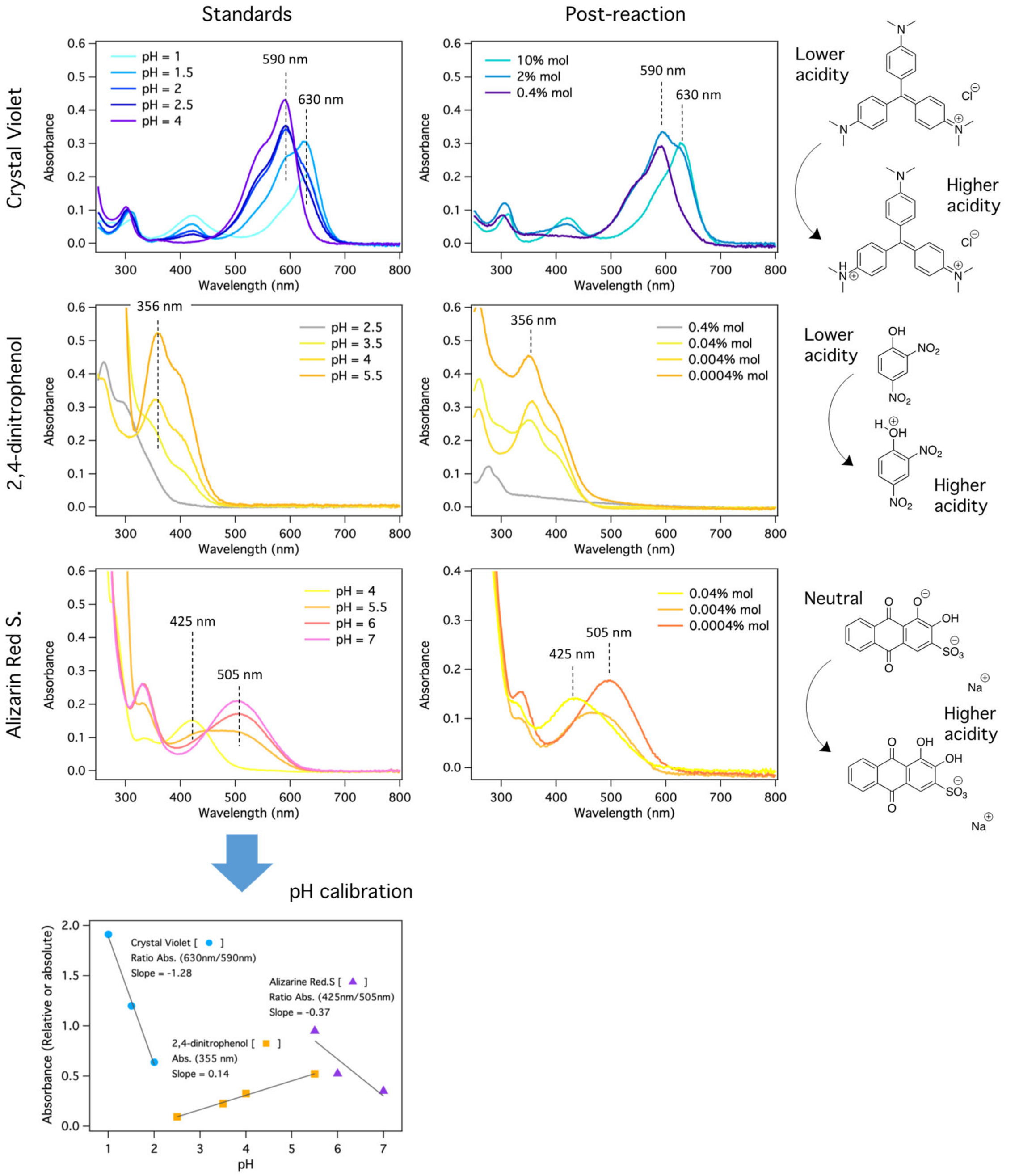

Figure S18. Left: UV-Vis calibrations for the probe molecules: crystal violet (1-2.5 pH), 2,4-dinitrophenol (2.5-4 pH), and alizarin red (4-7 pH). Bottom: UV–vis absorption values of crystal violet (blue markers), 2,4–dinitrophenol (yellow markers) and alizarin red (purple markers) along the acid range of the pH scale. Right: UV-Vis results for the pH measurements of the solutions with 0.0004% (alizarin red, 2,4-dinitrophenol), 0.004% (alizarin red, 2,4-dinitrophenol), 0.04% (alizarin red, 2,4-dinitrophenol), 0.4% (2,4-dinitrophenol, crystal violet), 2% (2,4-dinitrophenol, crystal violet), 10% (2,4-dinitrophenol, crystal violet). The colors of the UV-vis curves approximate the colors of the solutions.

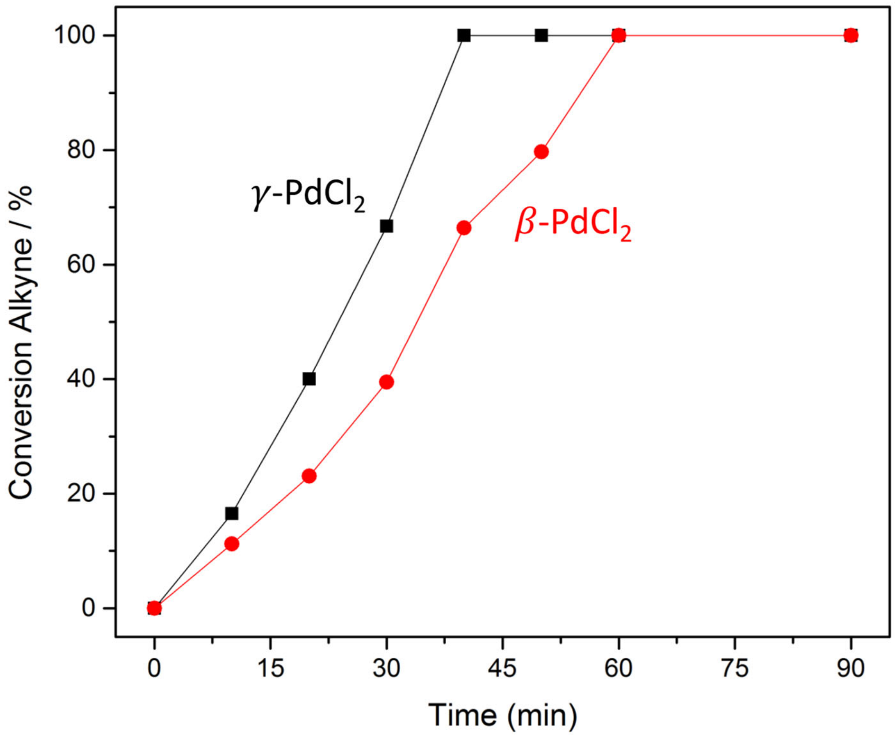

Figure S19. Conversion plots for the hydrogenation of **1** (0.5M in ethanol). Black lines: γ-$PdCl_2$, red lines: β-$PdCl_2$, 0.04 mol% of Pd, 30 ºC, 5 bar H_2.

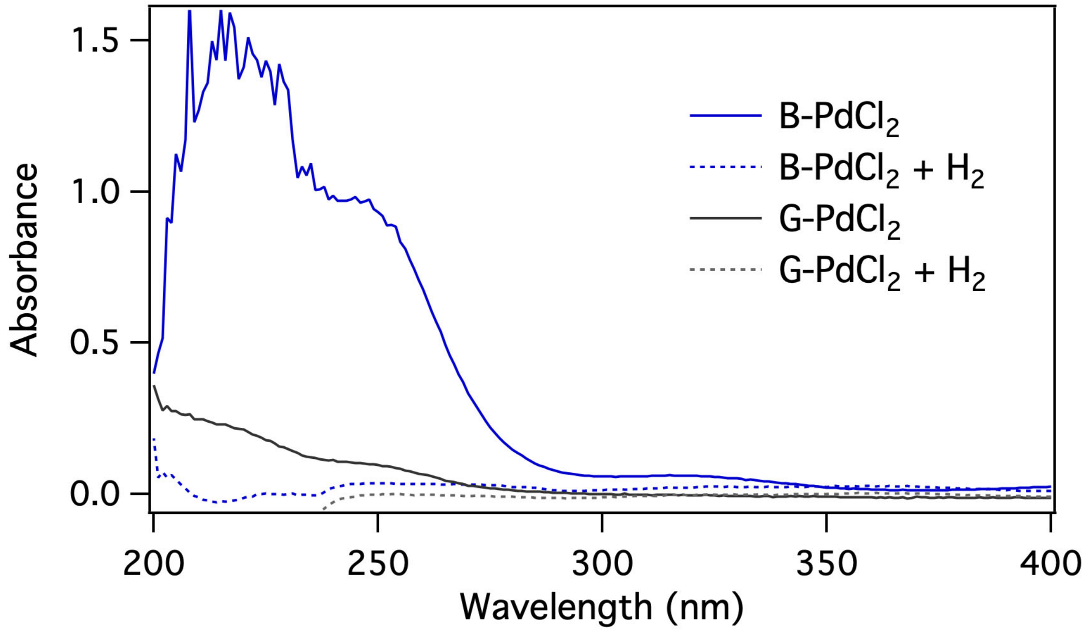

Figure S20. UV-Vis spectra of β-$PdCl_2$ and γ-$PdCl_2$ solutions in ethanol, before (solid lines) and after (dashed lines) placing H_2 (1 bar) in the cuvette. Blue curves: β-$PdCl_2$, black curves: γ-$PdCl_2$.

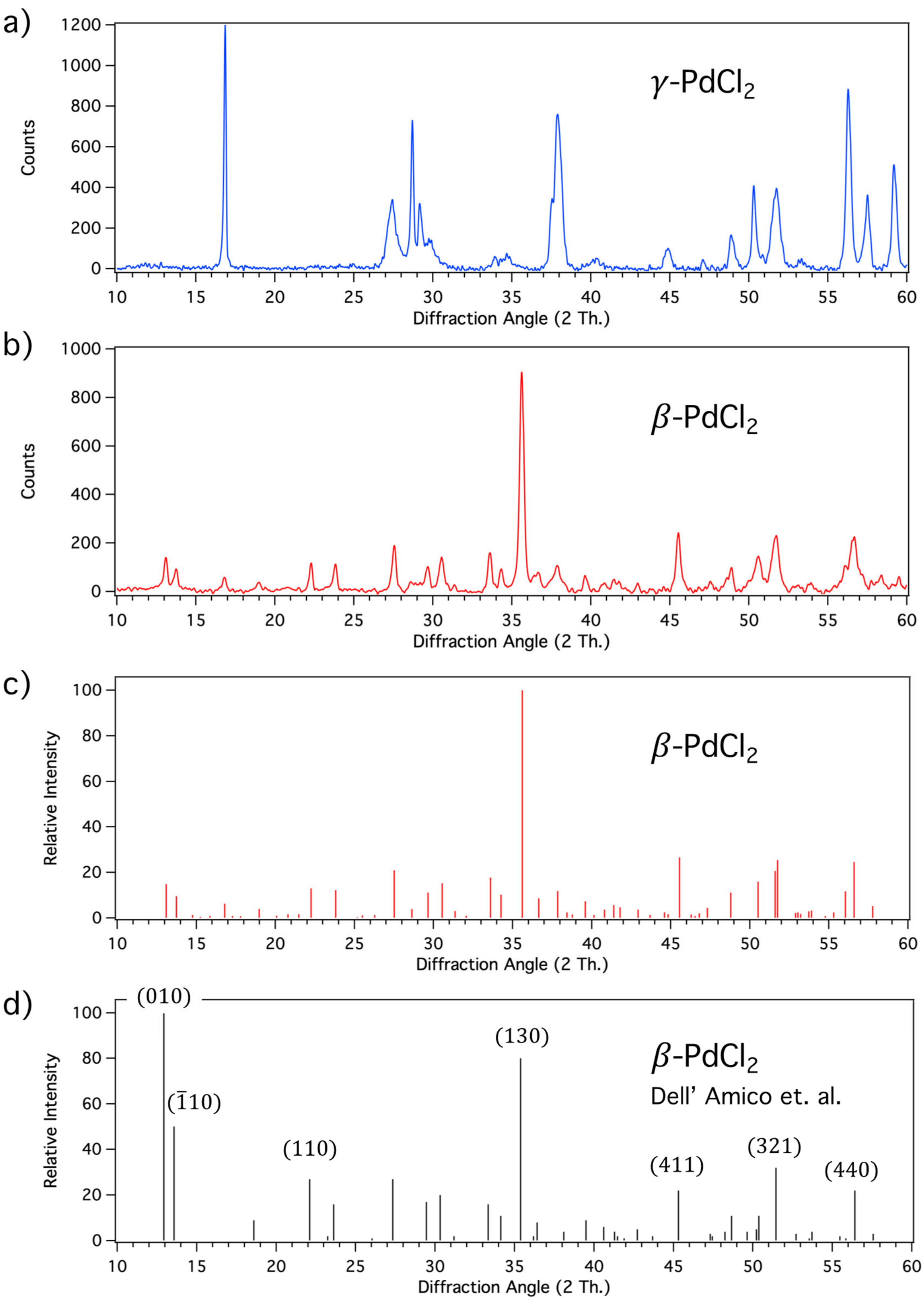

Figure S21. XRD spectra for a) commercial γ-$PdCl_2$, b) β-$PdCl_2$. XRD relative peak intensities for c) the synthesized β-$PdCl_2$ and d) the reported values for β-$PdCl_2$ in the literature,[S2] transformed from diffraction distances to diffraction angles. All XRD spectra were obtained with a Cu K(α) radiation source, 1.5406 Å wavelength.

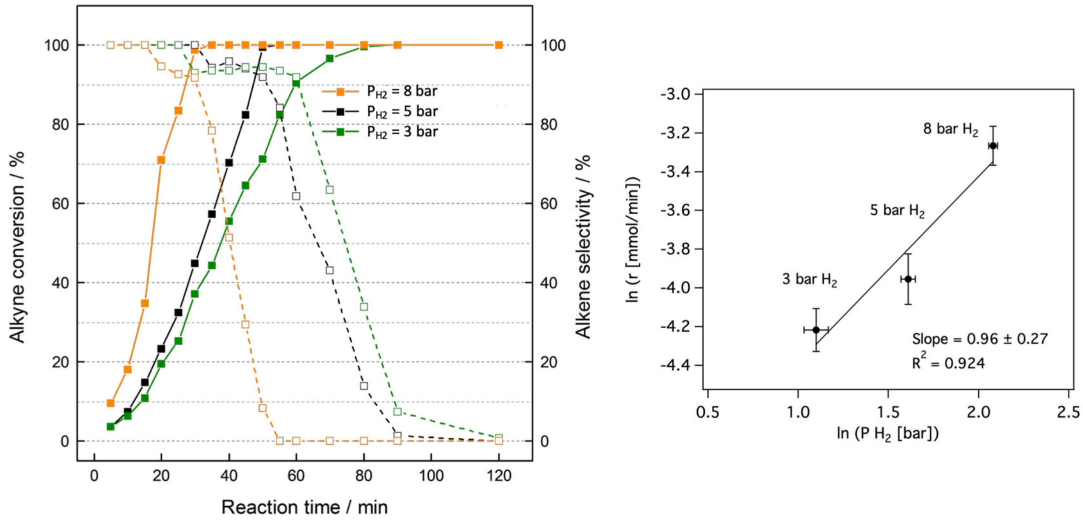

Figure S22. Left: Kinetic plot for the hydrogenation of **1** to **2** with 0.04 mol% of $PdCl_2$ (1 wt%) in EtOH (0.5M) under different pressure of H_2 at 30 ºC. The solid markers represent alkyne conversion and the hollow markers represent alkene selectivity; the balance is the corresponding alkane. Right: estimation of the reaction order for H_2. Error bars account for a 5% uncertainty in the y-axis and ± 0.2 bar in the x-axis.

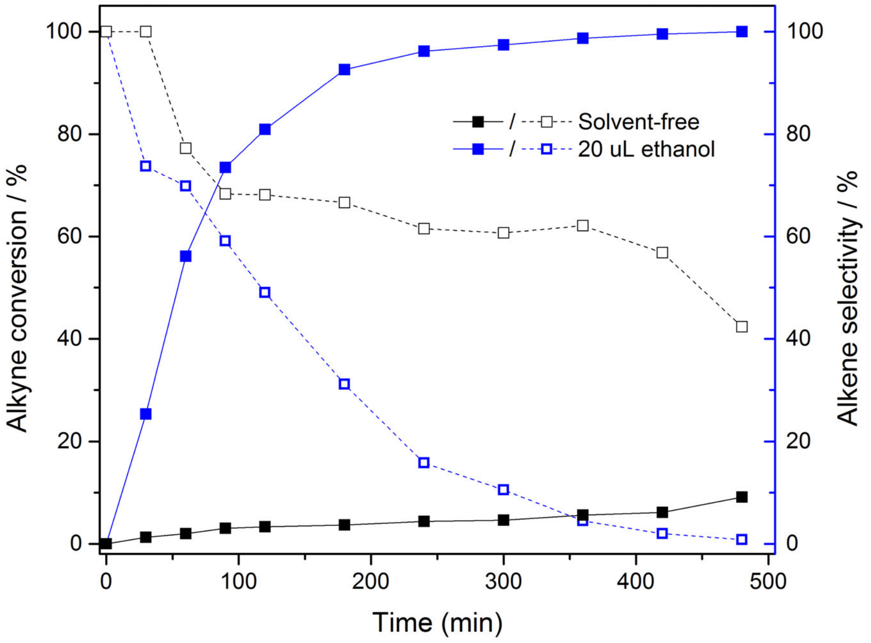

Figure S23. Kinetic plot for the hydrogenation of **1** to **2** with 0.0004 mol% of $PdCl_2$ in EtOH (0.5M) with 5 bar H_2 under: solvent-free (black curve) and solvent starved conditions (blue curve). Filled markers represent conversion, hollow markers represent selectivity.

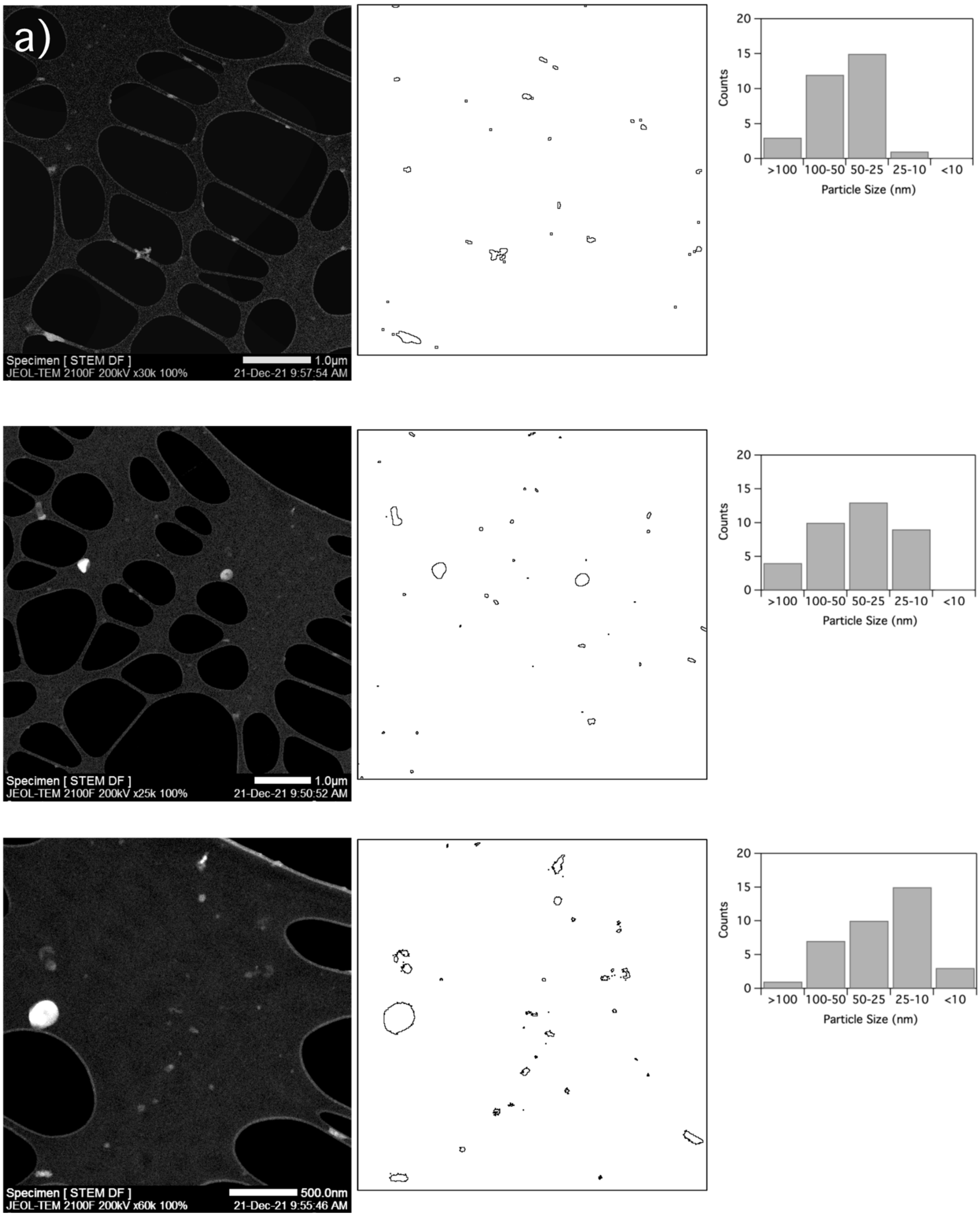
a)
Specimen [STEM DF]
1.0µm
JEOL-TEM 2100F 200kV x30k 100%
21-Dec-21 9:57:54 AM
Specimen [STEM DF]
1.0µm
JEOL-TEM 2100F 200kV x25k 100%
21-Dec-21 9:50:52 AM
Specimen [STEM DF]
500.0nm
JEOL-TEM 2100F 200kV x60k 100%
21-Dec-21 9:55:46 AM
Counts
>100
100-50
50-25
25-10
<10
Particle Size (nm)

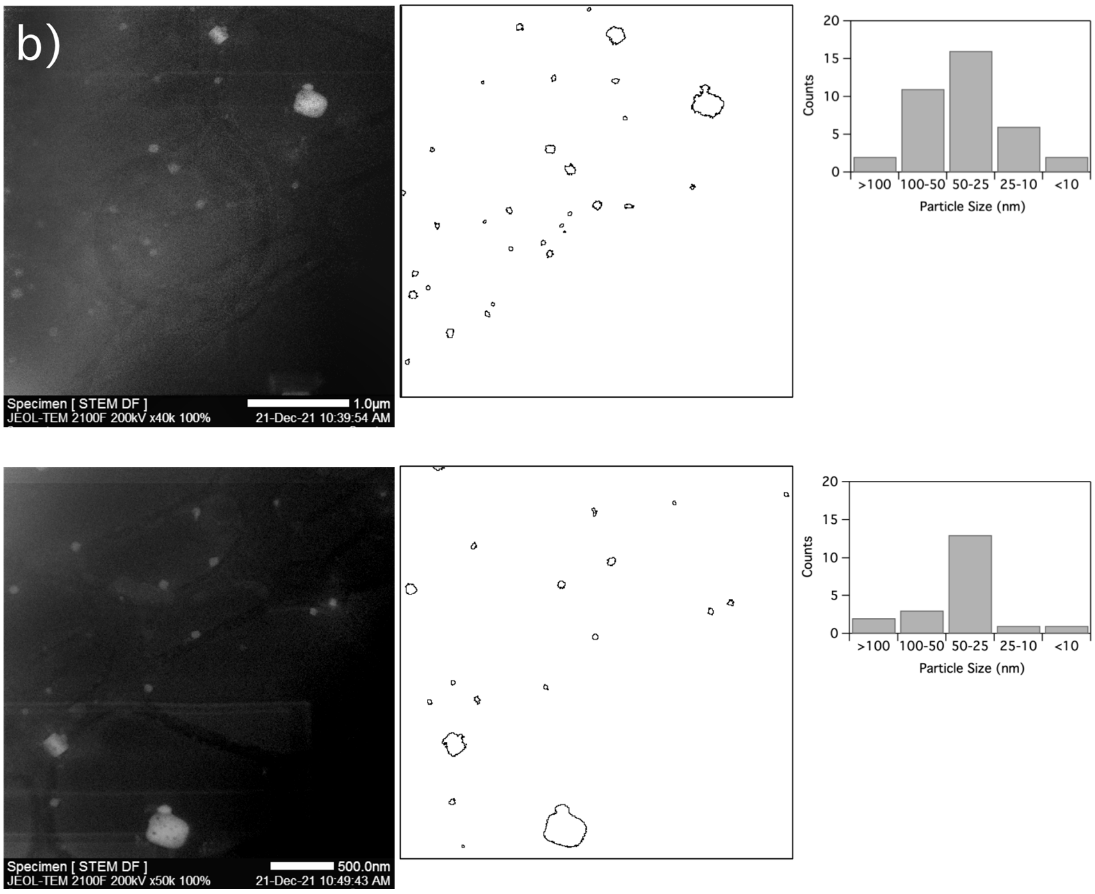
b)
Specimen [STEM DF]
1.0µm
JEOL-TEM 2100F 200kV x40k 100%
21-Dec-21 10:39:54 AM
Counts
Particle Size (nm)
>100
100-50
50-25
25-10
<10
Specimen [STEM DF]
500.0nm
JEOL-TEM 2100F 200kV x50k 100%
21-Dec-21 10:49:43 AM
Counts
Particle Size (nm)
>100
100-50
50-25
25-10
<10

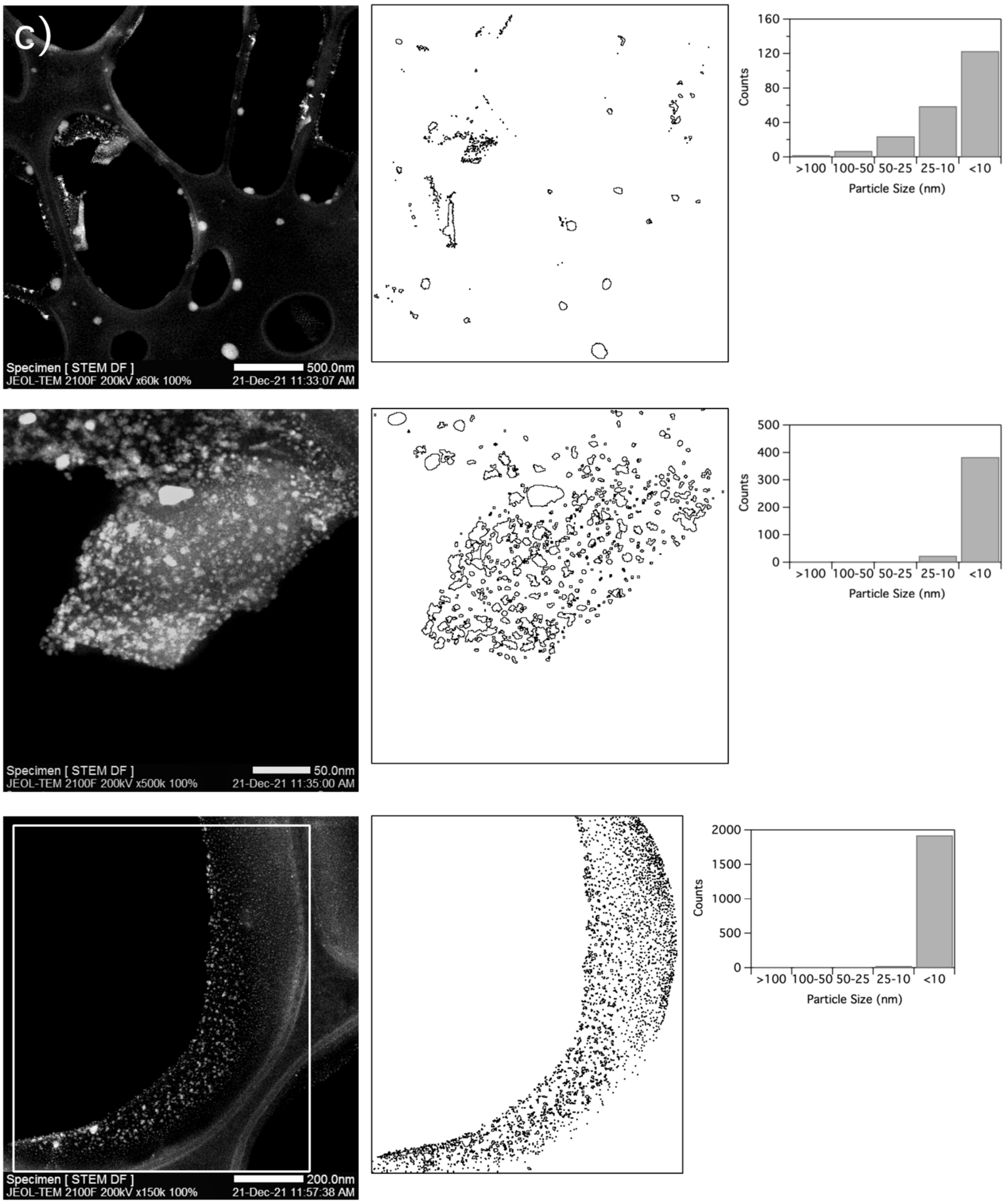

Figure S24. HR-TEM images and particle size distributions of a) 2 mol%, b) 0.4 mol% and c) 0.04 mol% samples. The images were processed to enhance contrast and reduce undesired glares, previous to the computer assisted particle outlining. The identification, mapping, and particle size estimation of the particles was performed in Image-J (Fiji package)[S1].

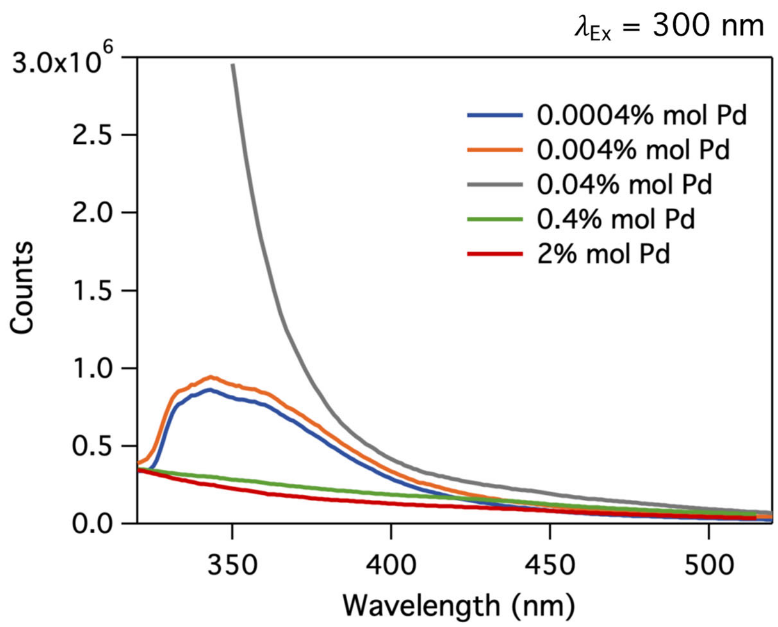

Figure S25. Fluorescence emission spectra ethanol solutions of 2 mol%, 0.4 mol%, 0.04 mol%, 0.004 mol%, 0.0004 mol% Pd loadings. At λ_{Ex} = 320 nm, lower counts were obtained, and lower excitation frequencies yielded no fluorescence signals.

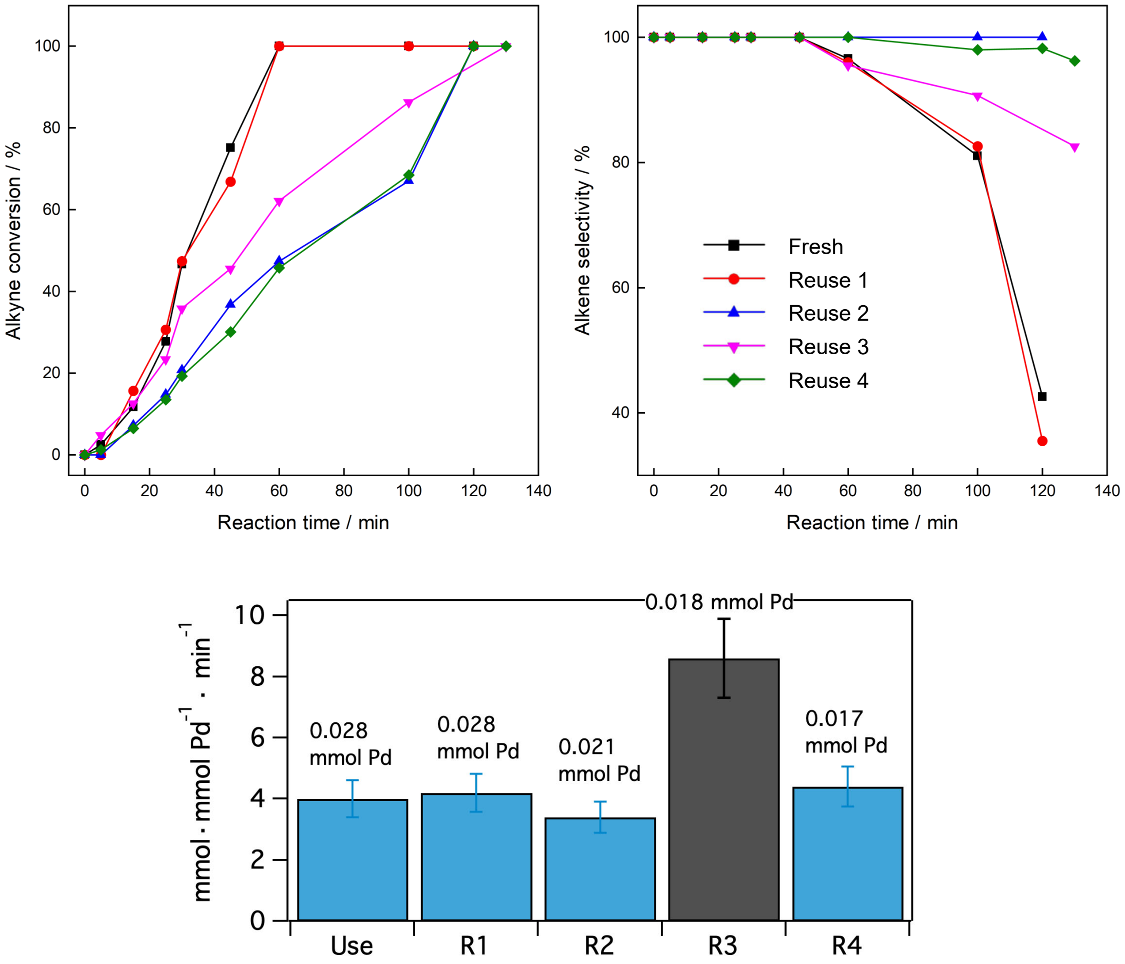

Figure S26. Top: kinetic plots for the hydrogenation of **1** to **2** with 0.04 mol% of $Pd_2(dba)_3$ on $MgCO_3$ in EtOH (0.5M) under 5 bars of H_2 at 30 ºC, representing alkyne conversion (left) and alkene selectivity (right) through different reuses. Selectivity balance corresponds to the alkane. Bottom: initial rates normalized by Pd amount in the reuse. The intrinsic activity remains fairly constant throughout the reuses, considering reuse 3 as an outlier.

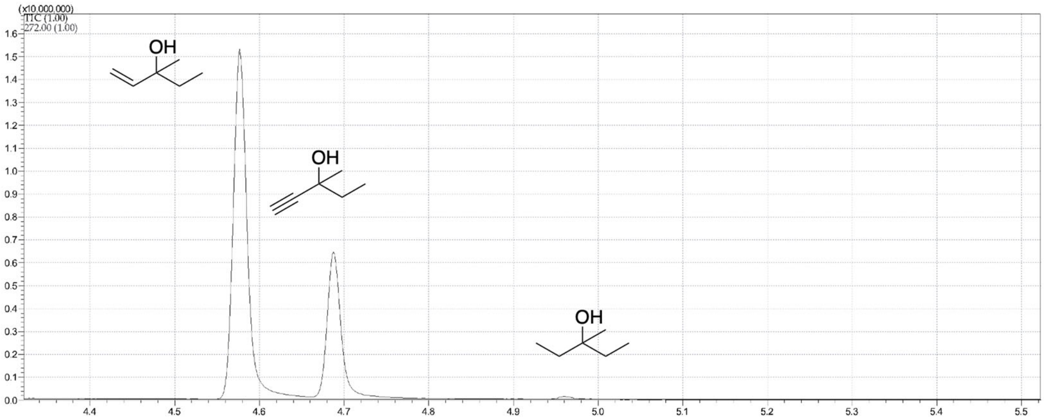
(x10,000,000)
TIC (1.00)
272.00 (1.00)
OH
OH
OH

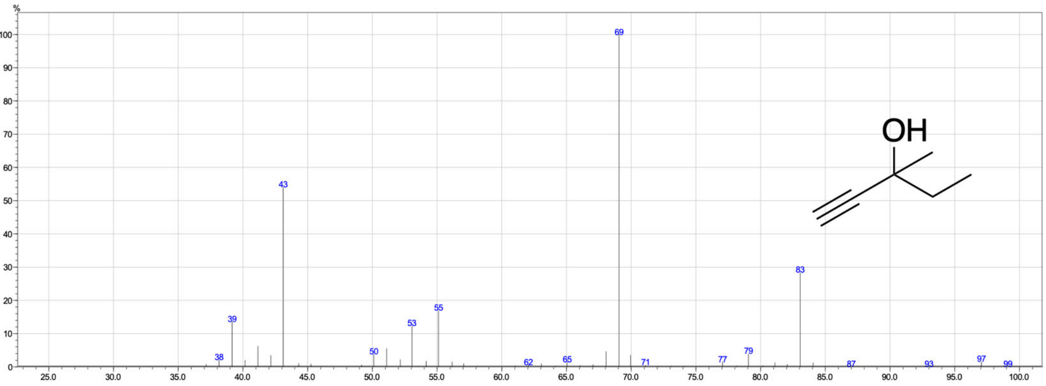
%
OH
69
43
83
39
55
53
38
50
62
65
71
77
79
87
93
97
99

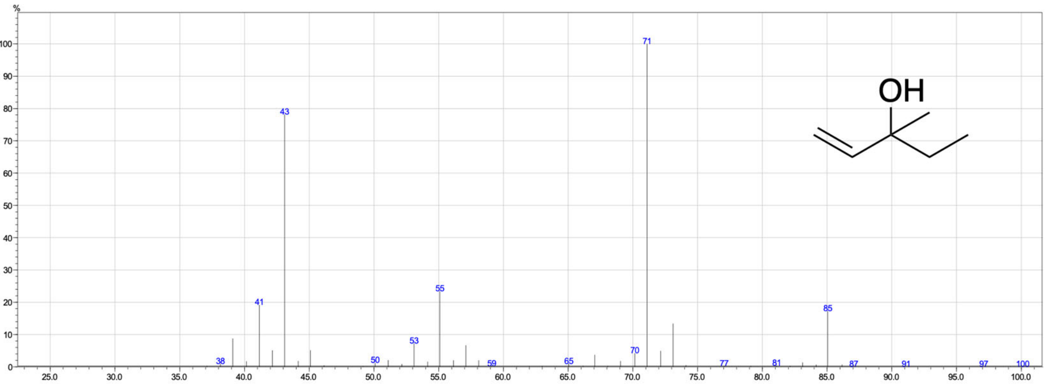
%
OH
71
43
41
55
85
53
38
50
59
65
70
77
81
87
91
97
100

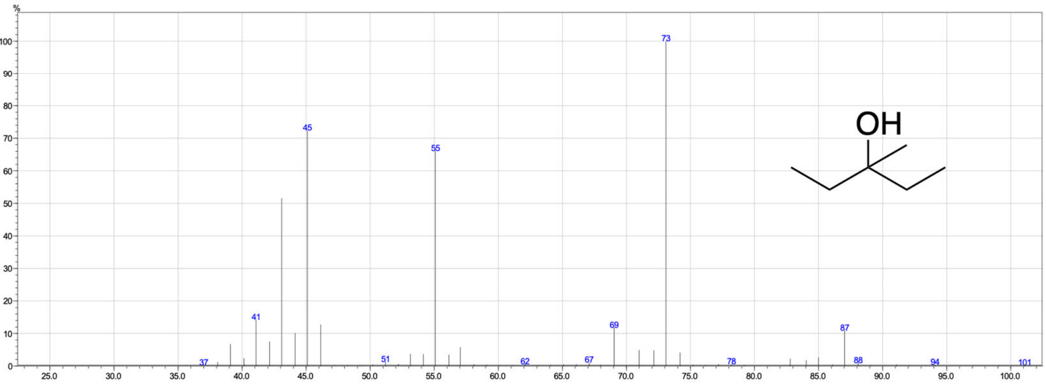
%
OH
73
45
55
41
69
87
37
51
62
67
78
88
94
101

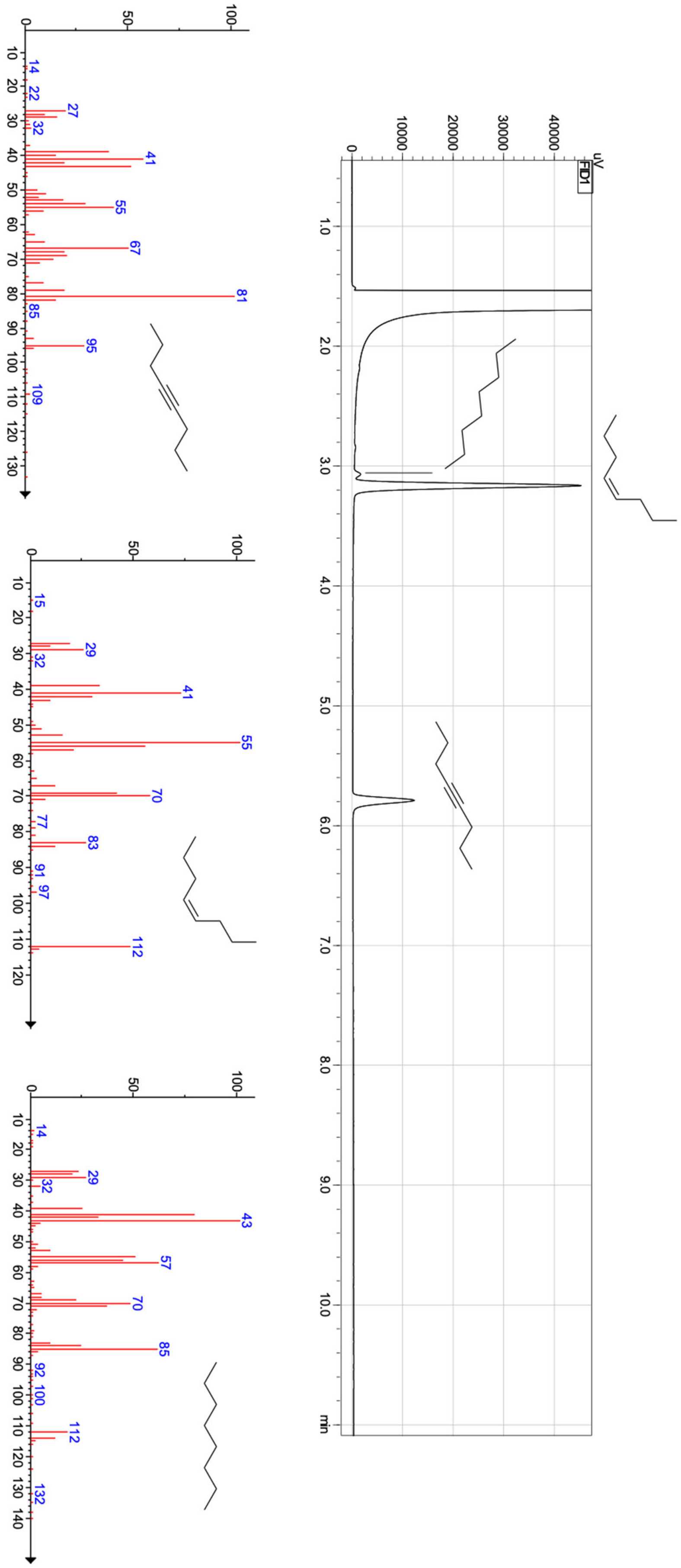
FID1
uV
min

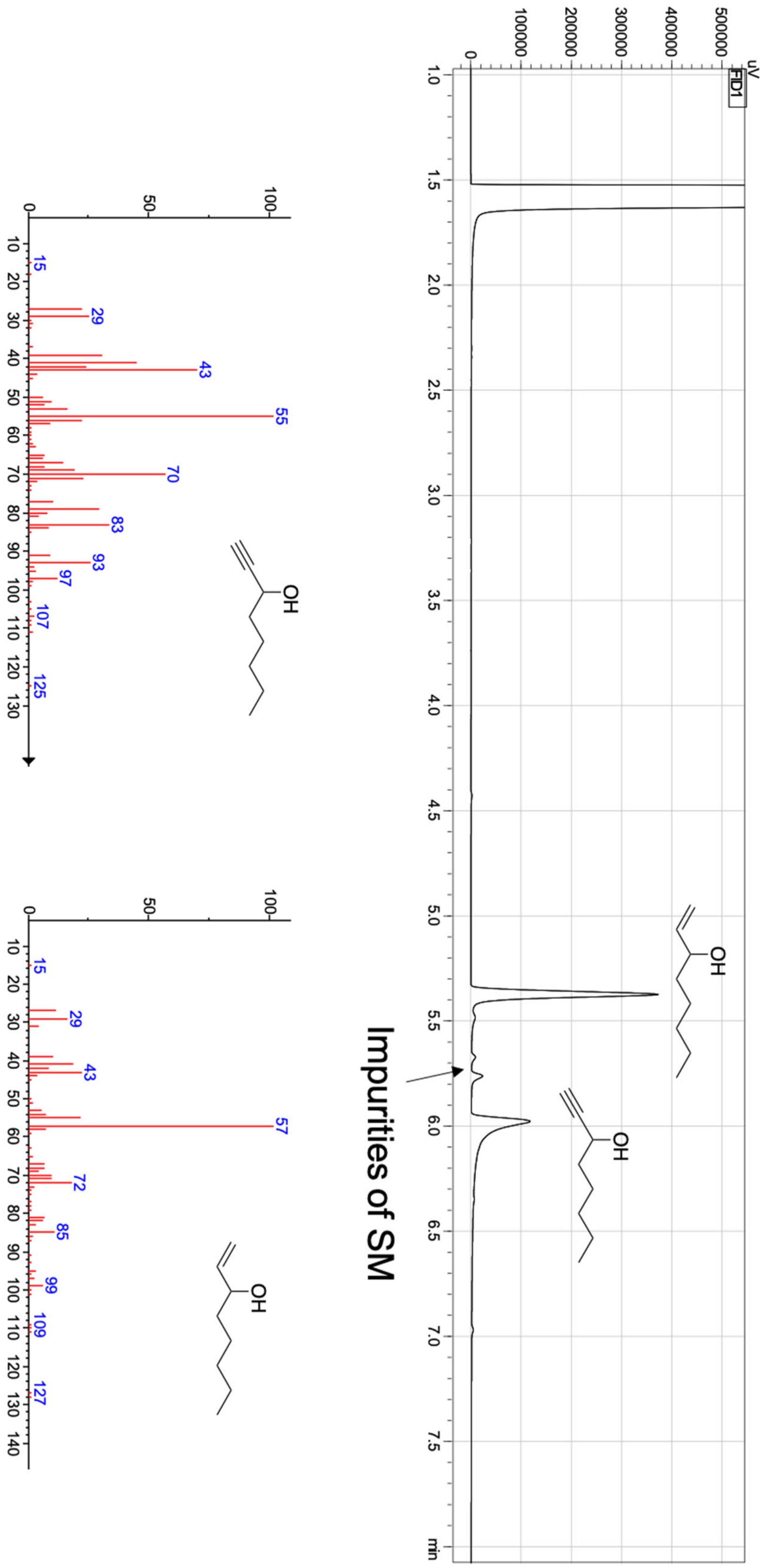
uV
FID1
Impurities of SM
OH
OH
OH
OH
min
15
29
43
55
70
83
93
97
107
125
15
29
43
57
72
85
99
109
127

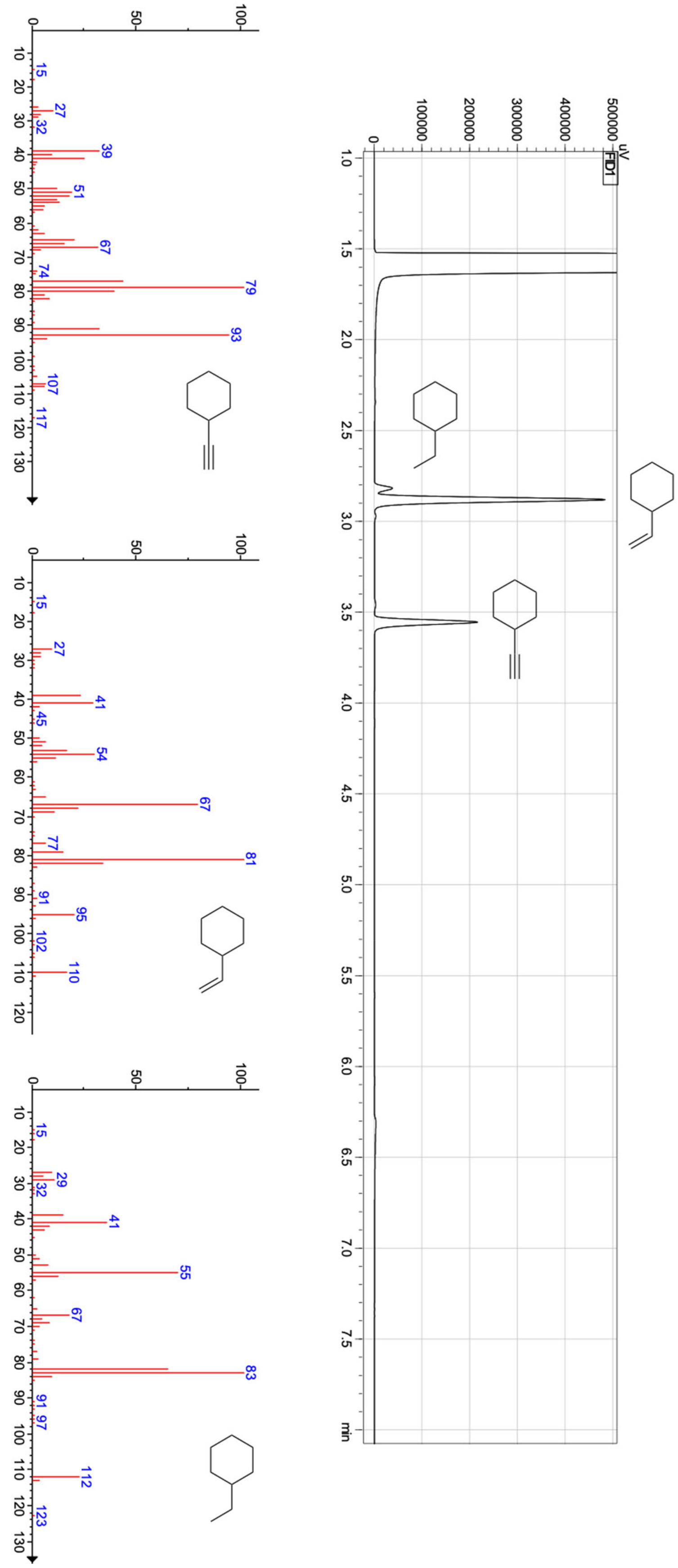

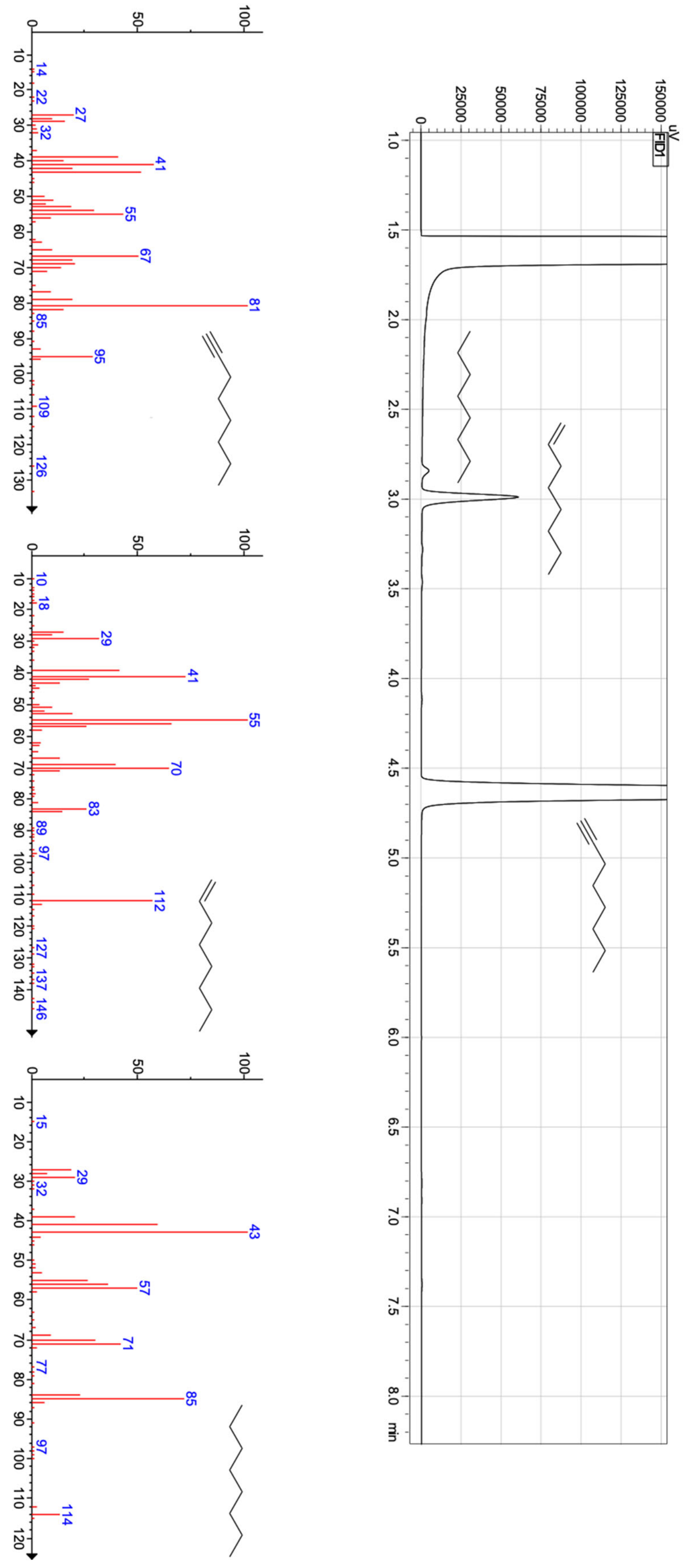
uV
FID1
min

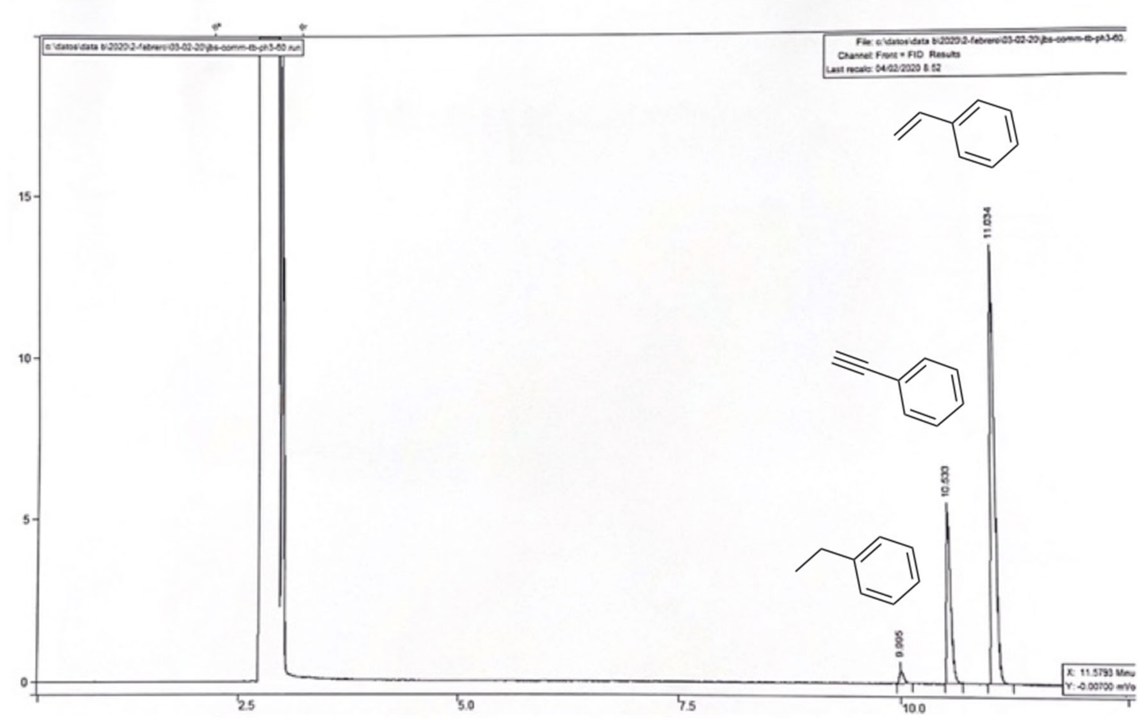
9.905
10.533
11.034
2.5
5.0
7.5
10.0

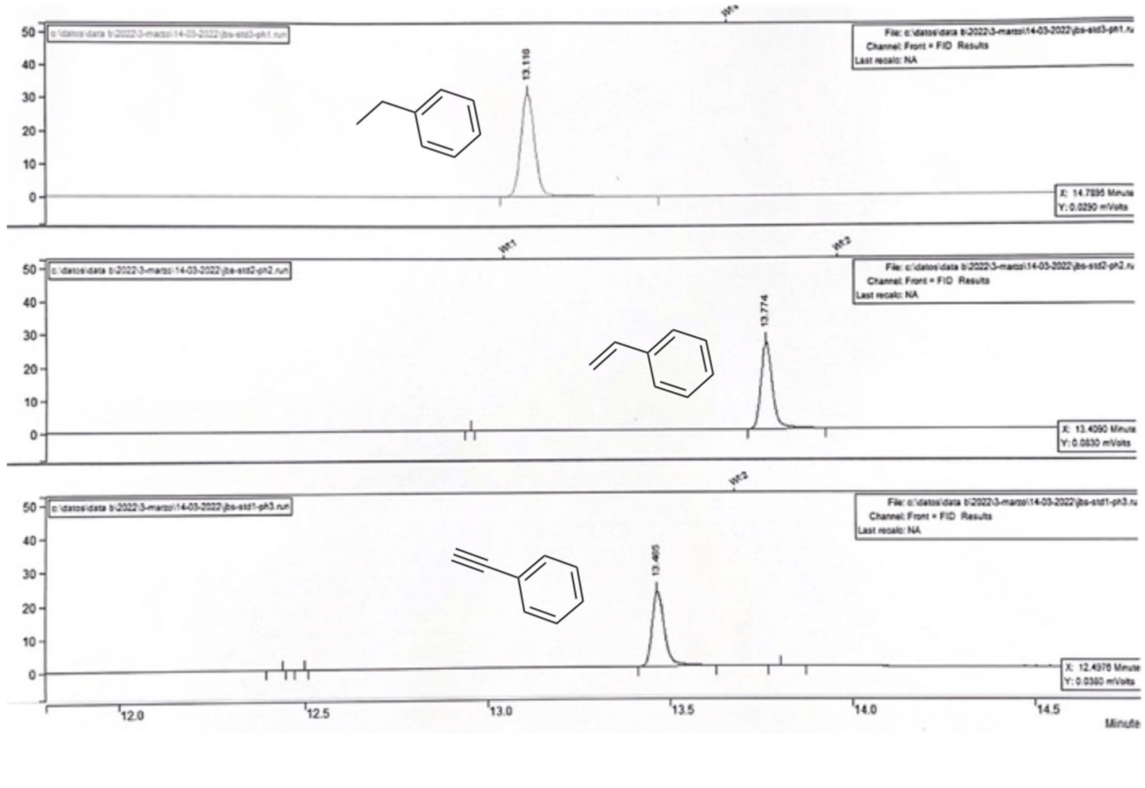
13.110
13.774
13.465
12.0
12.5
13.0
13.5
14.0
14.5
Minute

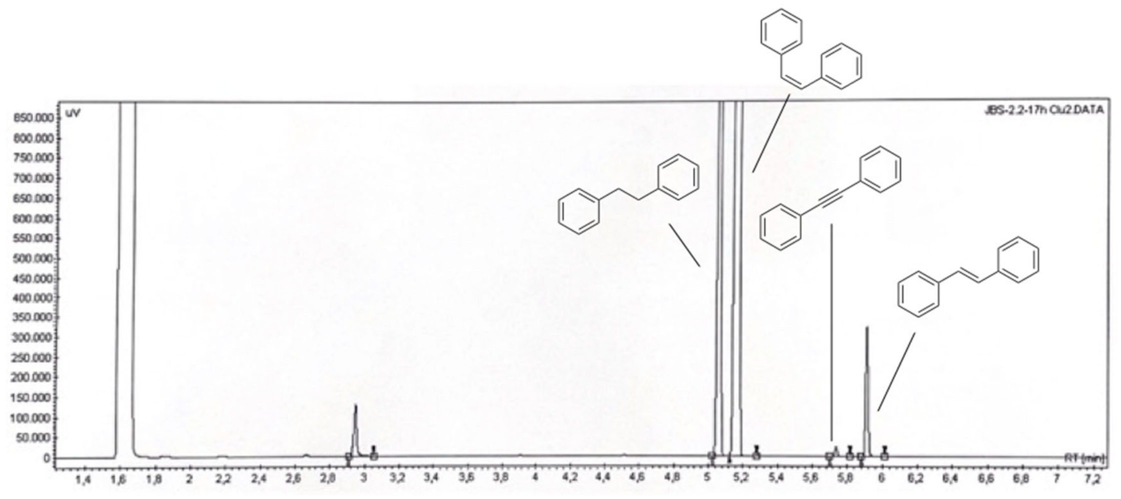
JBS-2.2-17h Clu2.DATA
uV
RT [min]

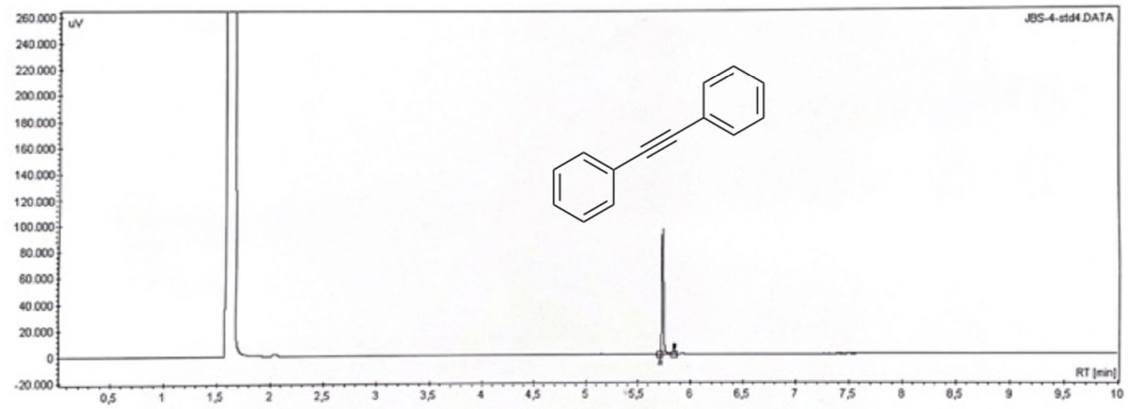
JBS-4-std4.DATA
uV
RT [min]

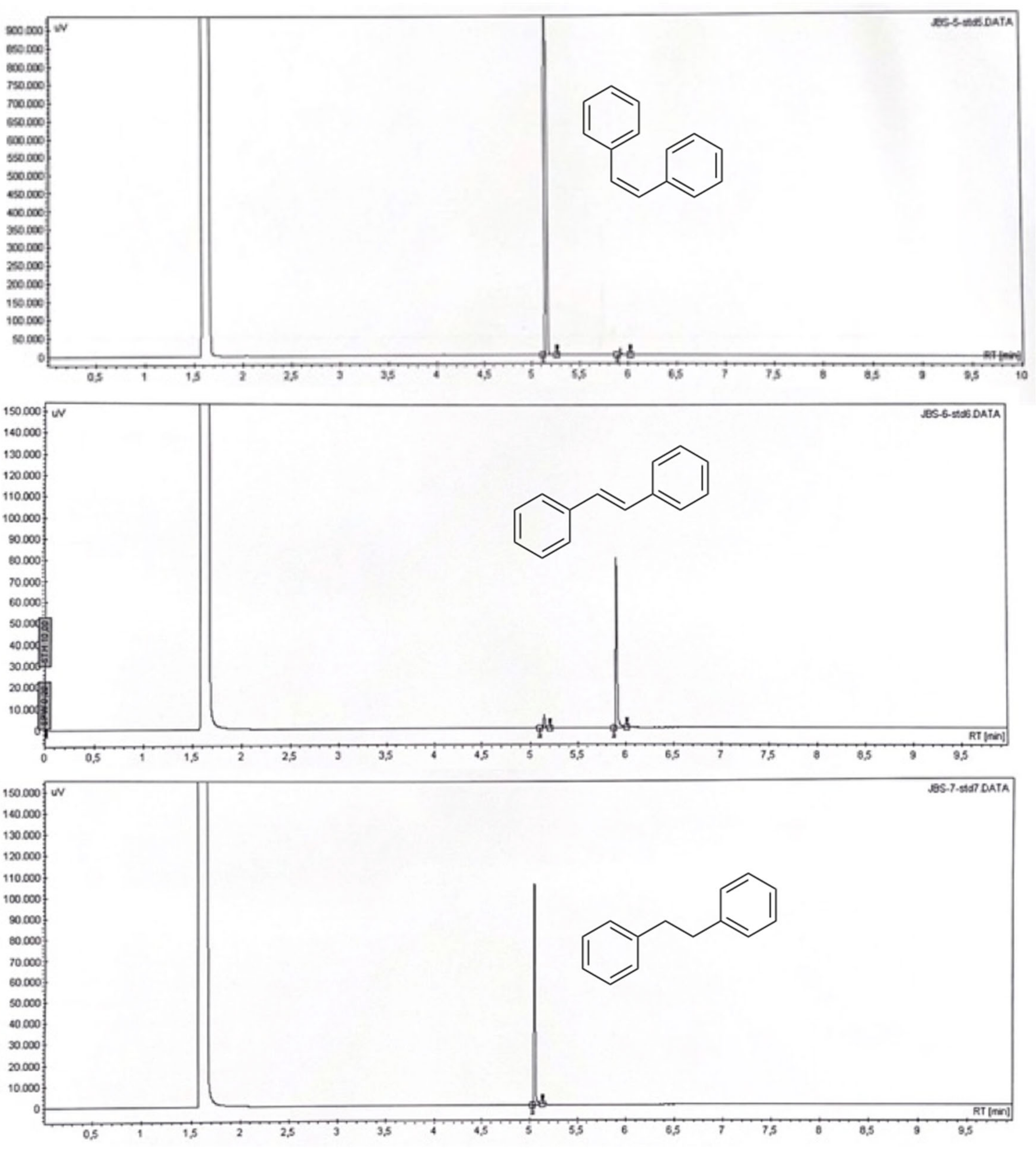

Figure S27. GC/GC-MS spectra, representative of the reactions of alkynes **1**, **3-8.**

Supporting Tables

Table S1. Bibliographic values for the intrinsic reaction rates of Pd-based catalysts during the hydrogenation of alkynes. (MOF: Metal Organic Framework, COF: Covalent Organic Framework, SAC: Single Atom Catalyst)

Catalyst	Substrate	Conditions	TOF (h^{-1})	Batch/Flow	Reference
Zr-MOF, sandwiched Pd NPs	Phenylacetylene	283 K, 5 bar H_2	13835	Batch	S3
Pd SAC on graphdiyne	Phenylacetylene	293 K, 2 bar H_2	6290	Batch	S4
Ni SAC on N-doped C	Acetylene	Pure acetylene flow, electrocatalysis	3848	Flow	S5
Low Pd content on COF-SO_3H	Phenylacetylene	298 K, 1bar H_2	3888	Batch	S6
Ni/Pd (70:30) colloidal particles	Phenylacetylene	323 K, 1 bar H_2	7092	Batch	S7
Lindlar	1-hexyne	293 K, 1 bar H_2	4900	Flow	S8
c-Pd/TiS	1-hexyne	293 K, 1 bar H_2	5500	Flow	S8
Lindlar	1-octyne	293 K, 1 bar H_2	4400	Flow	S8
c-Pd/TiS	1-octyne	293 K, 1 bar H_2	4700	Flow	S8
Lindlar	3-hexyne	293 K, 1 bar H_2	5400	Flow	S8
c-Pd/TiS	3-hexyne	293 K, 1 bar H_2	4900	Flow	S8
Lindlar	4-octyne	293 K, 1 bar H_2	5100	Flow	S8
c-Pd/TiS	4-octyne	293 K, 1 bar H_2	0	Flow	S8
Lindlar	Phenylacetylene	293 K, 1 bar H_2	4800	Flow	S8

c-Pd/TiS	Phenylacetylene	293 K, 1 bar H_2	4800	Flow	S8
Lindlar	MBY	303 K, 1 bar H_2	100	Flow	S9
Lindlar	MBY	303 K, 4 bar H_2	150	Flow	S9
c-Pd/TiS	1-hexyne	303 K, 1 bar H_2	1000	Flow	S9
c-Pd/TiS	1-dodecyne	303 K, 1 bar H_2	2100	Flow	S9
c-Pd/TiS	MBY	303 K, 1 bar H_2	1500	Flow	S9
c-Pd/TiS	3-hexyne	303 K, 1 bar H_2	1600	Flow	S9
c-Pd/TiS	4-octyne	303 K, 1 bar H_2	1400	Flow	S9

Table S2: UV–vis results and pH values of the different $PdCl_2$ solutions in water after 30 minutes under H_2. *The values are discarded for the pH calculations. **Extrapolated, out of calibration range.

pH Indicators	UV–Vis bands	Pd loadings (mol%)					
		10%	2%	0.40%	0.04%	0.004%	0.0004%
Crystal violet	Abs (590 nm)	0.184	0.334	0.290	-	-	-
	Abs (630 nm)	0.300	0.277	0.094	-	-	-
	Ratio (630/590)	1.634	0.829	0.326	-	-	-
2,4–Dinitrophenol	Abs (355 nm)	-	-	0.034*	0.259	0.363*	-
Alizarin red	Abs (425nm)	-	-	-	0.142	0.105	0.116
	Abs (505nm)	-	-	-	0.102	0.104	0.178
	Ratio (425/505)	-	-	-	1.389*	1.010	0.650
pH values	Experimental	1.20	1.83	2.22**	3.65	5.07	6.05
	Theoretical	1.28	1.98	2.68	3.68	4.68	5.68

References

(S1) Schindelin, J.; Arganda-Carreras, I.; Frise, E.; Kaynig, V.; Longair, M.; Pietzsch, T.; Preibisch, S.; Rueden, C.; Saalfeld, S.; Schmid, B.; et al. Fiji: An Open-Source Platform for Biological-Image Analysis. *Nat. Methods* **2012**, *9* (7), 676–682. https://doi.org/10.1038/nmeth.2019.

(S2) Belli Dell Amico, D.; Calderazzo, F.; Marchetti, F.; Ramello, S. Molecular Structure of [Pd_6Cl_{12}] in Single Crystals Chemically Grown at Room Temperature. *Angew. Chem., Int. Ed.* **1996**, *35* (12), 1331–1333.

(S3) Choe, K.; Zheng, F.; Wang, H.; Yuan, Y.; Zhao, W.; Xue, G.; Qiu, X.; Ri, M.; Shi, X.; Wang, Y.; et al. Fast and Selective Semihydrogenation of Alkynes by Palladium Nanoparticles Sandwiched in Metal–Organic Frameworks. *Angew. Chemie* **2020**, *132* (9), 3679–3686. https://doi.org/10.1002/ange.201913453.

(S4) Yin, X. P.; Tang, S. F.; Zhang, C.; Wang, H. J.; Si, R.; Lu, X. L.; Lu, T. B. Graphdiyne-Based Pd Single-Atom Catalyst for Semihydrogenation of Alkynes to Alkenes with High Selectivity and Conversion under Mild Conditions. *J. Mater. Chem. A* **2020**, *8* (40), 20925–20930. https://doi.org/10.1039/d0ta07705d.

(S5) Ma, W.; Chen, Z.; Bu, J.; Liu, Z.; Li, J.; Yan, C.; Cheng, L.; Zhang, L.; Zhang, H.; Zhang, J.; et al. π-Adsorption Promoted Electrocatalytic Acetylene Semihydrogenation on Single-Atom Ni Dispersed N-Doped Carbon. *J. Mater. Chem. A* **2022**, *8*. https://doi.org/10.1039/d1ta08002d.

(S6) Li, J. H.; Yu, Z. W.; Gao, Z.; Li, J. Q.; Tao, Y.; Xiao, Y. X.; Yin, W. H.; Fan, Y. L.; Jiang, C.; Sun, L. J.; et al. Ultralow-Content Palladium Dispersed in Covalent Organic Framework for Highly Efficient and Selective Semihydrogenation of Alkynes. *Inorg. Chem.* **2019**, *58* (16), 10829–10836. https://doi.org/10.1021/acs.inorgchem.9b01117.

(S7) Domínguez-Domínguez, S.; Berenguer-Murcia, Á.; Cazorla-Amorós, D.; Linares-Solano, Á. Semihydrogenation of Phenylacetylene Catalyzed by Metallic Nanoparticles Containing Noble Metals. *J. Catal.* **2006**, *243* (1), 74–81. https://doi.org/10.1016/j.jcat.2006.06.027.

(S8) Vilé, G.; Almora-Barrios, N.; Mitchell, S.; Lopez, N.; Pérez-Ramírez, J. From the Lindlar Catalyst to Supported Ligand-Modified Palladium Nanoparticles: Selectivity Patterns and Accessibility Constraints in the Continuous-Flow Three-Phase Hydrogenation of Acetylenic Compounds. *Chem.Eur. J.* **2014**, *20* (20), 5926–5937. https://doi.org/10.1002/chem.201304795.

(S9) Albani, D.; Shahrokhi, M.; Chen, Z.; Mitchell, S.; Hauert, R.; López, N.; Pérez-ramírez, J. Selective Ensembles in Supported Palladium Sulfide Nanoparticles for Alkyne Semi-Hydrogenation. *Nat. Commun.* **2018**, No. 9:2634, 1–11. https://doi.org/10.1038/s41467-018-05052-4.
